# Experimental investigation of training schedule on home-based working memory training in healthy older adults

**DOI:** 10.3389/fpsyg.2023.1165275

**Published:** 2023-04-28

**Authors:** Samantha J. Booth, Laura J. E. Brown, Jason R. Taylor, Gorana Pobric

**Affiliations:** ^1^Division of Psychology, Communication, and Human Neuroscience, School of Health Sciences, Manchester Academic Health Science Centre, The University of Manchester, Manchester, United Kingdom; ^2^Division of Psychology and Mental Health, School of Health Sciences, Manchester Academic Health Science Centre, The University of Manchester, Manchester, United Kingdom

**Keywords:** aging, cognitive enhancement, cognitive training, working memory, working memory training

## Abstract

**Introduction:**

The efficacy of working memory training (WMT) for cognitive enhancement in healthy older adults has been extensively investigated. Typically, WMT results in improved performance on the training task, but limited or no transfer of improvement to other cognitive tasks. Accordingly, there is a need to identify optimal intervention parameters to maximize training and transfer task effects of WMT. The current study aimed to investigate the effect of training schedule on training and transfer task performance of WMT in healthy older adults. A secondary aim was to examine the feasibility of participants performing the intervention online at home, unsupervised, and using their personal devices.

**Methods:**

Participants (*N* = 71; mean age: 66 years) completed sixteen WMT or active-control sessions over eight (distributed) or four (intensive) weeks. Adaptive verbal and spatial n-back tasks were used as the WMT tasks. We tested near transfer effects to a digit-span task and far transfer effects to an abstract relational reasoning task.

**Results:**

Participants successfully performed the cognitively demanding intervention using their own devices, online at home, and with minimal contact with the researcher. We observed a significant improvement in WMT task performance in the WMT group relative to active-controls, but no evidence of near or far transfer. Similar training effects were observed irrespective of the intensity of the training schedule.

**Discussion:**

Our results suggest that comparable benefits could be observed when using less intensive schedules that may be more easily accommodated into everyday life.

## 1. Introduction

Changes in cognitive functions over time in normal aging have been described by Zaninotto et al. (2018). One of the major concerns for healthy older adults is decline in cognitive function (Sabia et al., 2012). It is known that different domains of cognition decline at different rates (Wilson et al., 2002). Therefore, studying age-related cognitive performance in healthy participants is important to potentially mitigate such decline and develop early interventions (Plassman et al., 2010).

It is well understood that working memory (WM) declines with healthy aging (Chai et al., 2018). WM, defined as the ability to retain and manipulate material over a short period in service of cognition (Baddeley and Hitch, 1974), is critical for many higher-order cognitive activities, like following instructions (Jaroslawska et al., 2016), decision making (Del Missier et al., 2013), and reasoning (Cowan, 2014). Even slight declines in WM can impact everyday functioning and wellbeing significantly (Wilson et al., 2013). Accordingly, the development of therapeutic interventions that may prevent or delay the onset or progression of healthy age-related WM decline is desired (Prehn and Flöel, 2015).

Working memory is considered an essential executive function, important for the successful execution of goal-directed behavior (Miyake et al., 2000; Diamond, 2013). The role of WM in higher-order cognition has motivated the development of cognitive training programs, with the assumption that if successful, WMT (WM training) could result in benefits to cognition more widely (Shipstead et al., 2012; Soveri et al., 2017). Cognitive training can be defined as a course of regular cognitive activities designed to challenge certain cognitive abilities to maintain or improve cognition in healthy and/or clinical populations (Gobet and Sala, 2022). Cognitive training is hypothesized to facilitate neuroplasticity, which in turn might mitigate cognitive decline and/or enhance performance (Duda and Sweet, 2019). Training typically involves multiple sessions in which participants complete complex WM tasks to train WM-related mechanisms (e.g., maintenance, updating etc.: Nee et al., 2013) according to a pre-defined schedule (Hou et al., 2020). If WMT is to have a widespread reach, it is essential that it can be performed outside the lab in an unsupervised environment using participants’ devices.

Several meta-analyses of WMT in older adults have reported improved training task performance immediately post-training (Karbach and Verhaeghen, 2014; Nguyen et al., 2019; Sala et al., 2019). However, to be beneficial, cognitive enhancement should translate to improvement on other tasks within the same domain as the training task (near transfer, such as other WM tasks), on tasks which tap into other domains (far transfer, for example, spatial navigation, or prospective memory) and ultimately, to everyday functioning (Mewborn et al., 2017). However, meta-analyses of WMT in older adults have reported relatively small or no effects of near and far transfer (Nguyen et al., 2019; Sala et al., 2019).

A number of training protocol characteristics have been investigated to identify an optimal intervention procedure to maximize training and transfer effects following WMT (Wang et al., 2014). One such dimension is practice distribution, which reflects the intensity of the training schedule (Takeuchi et al., 2010). For example, the same number of hours of training may be clustered into a short period or distributed over a longer period. There is evidence from other cognitive fields that distributed learning is superior to massed learning (Cepeda et al., 2006). This is known as the spacing effect and has been observed during various learning instances, like second language learning (Kim and Webb, 2022). A meta-analysis of WMT studies in healthy older adults found that distributed training (<3 sessions per week) was more effective than intensive training (>3 sessions per week) (Hou et al., 2020). This suggests that the spacing effect might apply to WMT in healthy older adults. However, limited conclusions can be drawn from this meta-analysis. For instance, the authors were unable to control for other intervention characteristics (e.g., dose) when investigating the impact of training schedule given that classical meta-analytical methods generalize over different study protocols (Wischnewski et al., 2021).

Single studies that directly compare the effectiveness of different training schedules have provided mixed results for the existence of a spacing effect with WMT. Specifically, some studies report that distributed training results in greater training and/or transfer effects relative to intensive training (young/middle-aged adults: Penner et al., 2012; children: Wang et al., 2014), whilst others report no impact of training schedule on training and transfer effects (young adults: Linares et al., 2019; older adults: Jaeggi et al., 2020). Only one study has investigated the effect of WMT training schedule in healthy older adults (Jaeggi et al., 2020). In this study participants trained twice per day, once per day, or every other day for twenty sessions. They observed improvement on the training and transfer tasks from pre- to post-training in each WMT group, but no difference in efficacy between the training schedules. This suggests that there is no impact of training intensity on WMT success in healthy older adults, at least with the range of schedules tested (Jaeggi et al., 2020).

For potential future applications of WMT, it is important to consider the practicality of the training schedule (Penner et al., 2012). Performing multiple sessions per day (as in Jaeggi et al., 2020) may result in cognitive fatigue and stress or be too demanding and unrealistic, which in turn might impact on compliance, adherence, and acceptability for the intervention (Penner et al., 2012; Hou et al., 2020). With this in mind, additional research using less intensive training schedules is important to advance the understanding of spacing effects in healthy older adults. A study in healthy young/middle-aged adults showed that a more distributed training schedule of two sessions per week for 8 weeks led to superior training and transfer effects relative to a more intensive schedule of four sessions per week for 4 weeks (Penner et al., 2012). The aim of the current study was therefore to determine whether a similarly distributed training schedule (of two sessions per week over 8 weeks) could result in enhanced training and transfer effects in healthy older adults compared to an intensive schedule of four sessions per week over 4 weeks. A secondary aim was to examine the feasibility of participants performing the intervention online at home, unsupervised, and using their own devices. The present study was defined as an efficacy study as the objective was to investigate the effect of WMT on cognitive performance relative to an active-control group (Green et al., 2019). We expected the WMT groups to show greater improvement in training and transfer task performance relative to active-controls. Moreover, given the proposed spacing effect during learning, we predicted greater improvement on the training and transfer tasks in the distributed-WMT group relative to the intensive-WMT group.

## 2. Materials and methods

### 2.1. Participants

Volunteers were recruited through community platforms in the Manchester (England) area including online advertisements in community newsletters, poster advertisements around the University of Manchester, and in-person advertisements at community groups (e.g., Age Friendly coffee mornings). All volunteers were required to meet the eligibility criteria in Table 1. The literature suggests that WM peaks during an individual’s early twenties and then shows a gradual decline across adulthood (Brockmole and Logie, 2013). Similar to other WM-related studies in older adults (Fournet et al., 2012; Atkinson et al., 2018; Matysiak et al., 2019; Reinhart and Nguyen, 2019), eligibility was restricted to people aged 55 years and above.

**TABLE 1:** Eligibility criteria.

Eligibility criteria
1. Able to provide written informed consent.
2. Aged 55 + years.
3. Normal or corrected-to-normal vision.
4. Fluent English speaker.
5. No severe non-correctable visual impairments.
6. No history of alcohol or substance abuse or dependency.
7. No history of neurological conditions (e.g., Dementia, Parkinson’s disease).
8. No history of psychiatric conditions [e.g., depression (if not in remission), schizophrenia].
9. Not color-blind.

Participants were allocated to one of four groups: distributed-WMT, intensive-WMT, distributed-active-control, or intensive-active-control. There is insufficient existing literature to enable a power analysis on the schedule (distributed/high-intensity) effect. Therefore, we powered the study based on the treatment (training/control) effect. Meta-analyses of WMT in older adults have reported medium-to-large effect sizes on the training task (Karbach and Verhaeghen, 2014; Nguyen et al., 2019; Sala et al., 2019). G*Power^1^ software for statistical power analysis and sample size calculations was used with a partial eta squared of 0.06 (medium). With a sample size of *N* = 34 (WMT: *n* = 17; active-control: *n* = 17) we had 80% power to detect an effect of this size on the treatment (WMT/control) outcome measure with an alpha of 0.05 (default correlation amongst repeated measures, 0.5). Therefore, we aimed to recruit 34 participants to each schedule (total: *N* = 68). Participants received a £40 shopping voucher, or a pro-rata amount if they withdrew early or were instructed to cease participation due to non-compliance with the study requirements (see section “2.4. Participant adherence and exclusions”). Seventy-nine participants started the study [mean age: 65.89 years ± 6.78 (55–85 years); male/female: 21/58].

### 2.2. Procedure

**FIGURE 1:**
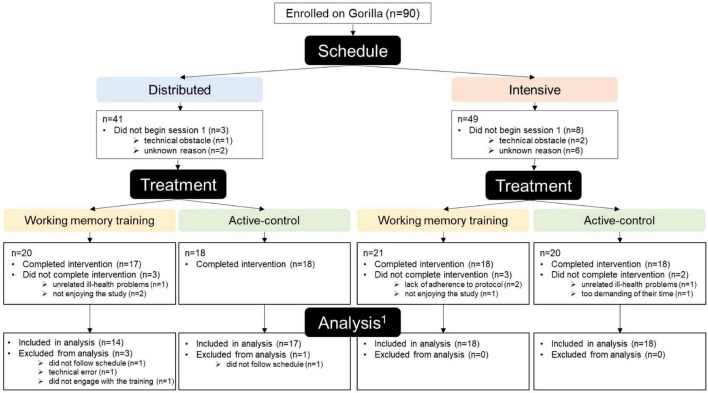
Flow diagram of progress through the phases of the study (^1^The number of participants eligible for inclusion in each analysis varies slightly, see section “2.4. Participant adherence and exclusions”).

This study was approved by the University of Manchester Research Ethics Committee (Ref: 2020-8907-16162) and was conducted following the 1964 Helsinki declaration. All participants provided informed consent using an online consent form completed at the beginning of their first session. Figure 1 illustrates progress through the study from enrolment to analysis, including attrition information for each group. A flowchart of the participant journey can be found in the (Supplementary Figure 1). After registering an interest in taking part, participants were allocated to a training schedule (distributed/intensive) on an alternating^2^ basis and enrolled onto the experimental platform Gorilla.^3^ On commencing the first session, participants were informed of the schedule they were allocated to and subsequently randomly allocated by Gorilla to the WMT or active-control group with a 1:1 ratio.

Participation involved 16 training sessions. Following Penner et al. (2012), under the distributed schedule participants in the WMT and active-control groups were instructed to train two times per week for 8 weeks. Under the intensive schedule participants in the WMT and active-control groups were instructed to train four times per week for 4 weeks. To prevent fatigue and equivalence between the two schedules, participants on the distributed schedule were instructed to have a space of 2 days between their two sessions per week, with a 5-day gap before the next week’s training. For example, if they started their first session on a Tuesday, they would train on a Tuesday and Thursday each week for 8 weeks. Participants on the intensive schedule were instructed to perform their four sessions per week on consecutive days, with a 4-day gap before the next week’s training. For instance, if they started their first session on a Tuesday, they would train on a Tuesday, Wednesday, Thursday, and Friday each week for 4 weeks. Timed delays between sessions were implemented by Gorilla (e.g., if a participant in the distributed group trained on a Tuesday they would not be able to access the next training session until Thursday). The spacing was implemented to ensure that (1) participants would not perform all their training tasks for 1 week on a single day, and (2) participants in the distributed group would not train on, for instance, Saturday/Sunday in week 1, then Monday/Tuesday in week 2, as this would reflect the intensive schedule. A figure illustrating the two training schedules can be found in the (Supplementary Figure 2).

Participants were asked to undertake each session in a quiet environment free of distractions. They were encouraged to perform each session at the same time of day, though this was not a strict requirement. Each session consisted of a verbal and a spatial WMT/active-control task. Within each group, the order that these tasks were performed was counterbalanced, with half of each group starting session one with the verbal task, and the other half with the spatial task. This order was switched at each session.

**FIGURE 2:**
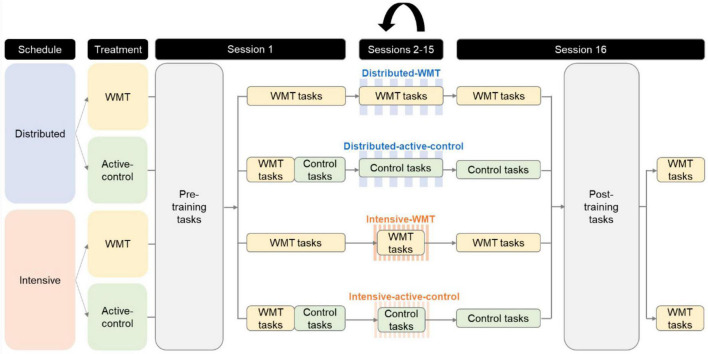
Illustration of the study procedure.

In session 1 (S1), participants performed three cognitive tasks (referred to as pre-training tasks in Figure 2) that collected baseline measures on near and far transfer tasks (∼15 min). This was followed by a 5-min break and then their first training session (∼20 min). In sessions 2–15, participants underwent ∼20 min of training per session. Participation concluded with session 16 (S16), where the final training task (∼20 min) was followed by a 5-min break, post-training measures of near and far transfer (referred to as post-training tasks in Figure 2), and an optional questionnaire (∼15 min). It was made clear to participants that once they pressed start on each task it would run automatically without the option to pause it. Due to the nature of the tasks (e.g., button presses), participation was restricted to a laptop and/or desktop computer using a feature on the Gorilla platform.

### 2.3. Cognitive tasks

#### 2.3.1. WMT tasks

**FIGURE 3:**
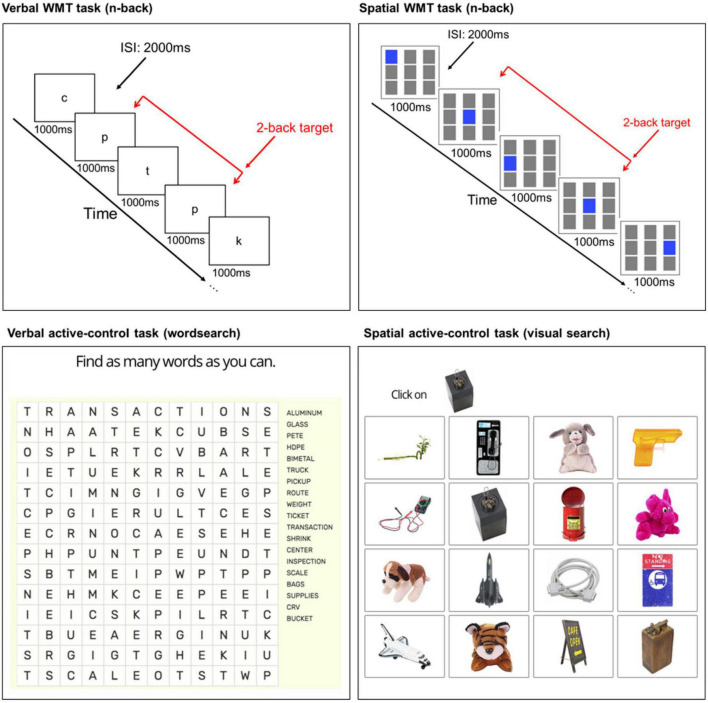
Illustration of the WMT and active-control tasks. Verbal **(top left)** and spatial **(top right)** n-back WMT tasks at load level two (2-back). Example verbal **(bottom left)** and spatial **(bottom right)** active-control tasks. ISI, inter-stimulus interval.

In a review paper on cognitive training methodologies, Simons et al. (2016), give clear recommendations for the design of training programs. One of those is adaptive training. In adaptive training, task difficulty increases as performance improves. It is believed that this challenges each participant to their limit and rules out the possibility that a lack of improvement could reflect an insufficiently demanding training procedure. Numerous studies have found superior training effects when training task difficulty was adaptive (e.g., Klingberg et al., 2002; Lövdén et al., 2010; Anguera et al., 2013; Brehmer et al., 2016). Therefore, an adaptive n-back paradigm (Figure 3) was used as the WMT task as it is a well-known paradigm that challenges WM (Sweet, 2011) and has been successfully used to train cognitive performance within our lab (Garg et al., 2022) and other labs (e.g., Jaeggi et al., 2008; Li et al., 2020). Two variants of the task (verbal/spatial) were used to facilitate task diversification.

A modified version of the adaptive n-back training task used by Jaeggi et al. (2008) was programmed in Gorilla.^4^ The verbal n-back used visual-verbal stimuli, comprised of eight lowercase English consonants (c, g, h, k, p, q, t, and w), presented on screen at a rate of one every 3 s (stimulus: 1000 ms; inter-stimulus interval: 2000 ms). For the spatial n-back, a 3 × 3 grid of squares were presented. Every 3 s one of the nine grids illuminated blue (stimulus) for 1000 ms, followed by a 2000 ms inter-stimulus interval.

Within each block, there were 20 + “n” trials (“*n*” = additional screens at the start of the sequence needed to create the particular n-back level, e.g., the first two screens cannot contain a target at 2-back). Six out of the 20 critical screens contained a target (where the letter or position of the illuminated square on the screen matched the one from “n” steps earlier) and the other 14 screens contained non-targets (i.e., non-repeats of the letter or illuminated square presented “n” steps earlier). The six target screens could appear in any of the n + 1 to n + 20 positions. The stimuli were arranged in a pseudo-randomized order, where the position of the targets changed in each block.

Participants were instructed to respond as quickly and accurately as possible with a spacebar press whenever the current stimulus was the same (verbal) or in the same position (spatial) as the one presented “n” steps back in the sequence (“*n*” = load level 1, 2, 3 etc.). No responses were required for non-targets. For example, at load level 1 (1-back), targets were defined as a stimulus that matched the letter (verbal) or position (spatial) of the stimulus immediately preceding it. At 2-back, participants responded if the stimulus matched the letter (verbal) or position (spatial) of the stimulus that was presented two trials before, and so on.

During each session, a practice 1-back block with accuracy feedback provided at each trial was used to enable participants to understand the task requirements. Participants could re-visit the task instructions, and repeat the practice block, as many times as they wanted before moving on to the training blocks. The task started with two 1-back blocks, followed by a 2-back block. From the third block, the task followed an adaptive staircase procedure to ensure that participants were always training at a challenging level.^5^ If overall accuracy was ≥90%, the level of the next block was increased by one. If overall accuracy was ≤70%, the level of the next block was decreased by one. Otherwise, the level stayed the same. Overall accuracy in each block was calculated from the last 20 screens (i.e., n + 1 to n + 20). A 15 s instruction screen before each block informed participants what n-back level they would be performing. Both n-back tasks comprised eight blocks, with a 60 s break after four blocks. Each task lasted ∼10 min (total: ∼20 min). The mean n-back level reached over the last five of the eight blocks served as the outcome measure.

#### 2.3.2. Active-control tasks

Verbal (wordsearch, ∼10 min) and spatial (visual search, ∼10 min) control tasks were presented at each active-control session (Figure 3). The wordsearch task had four rounds. In each round, a novel wordsearch was presented on screen. Participants had 2 min to locate and mark as many words as possible. In each trial of the visual search task, a 4 × 4 array of objects was presented with a target object presented at the top of the screen. Participants were tasked with locating and clicking on the target object^6^ from the array. A different 4 × 4 array and target object was presented at each trial. Further task details are reported in the Supplementary material.

#### 2.3.3. Near transfer tasks

Computerized digit-span forward (DSF) and backward (DSB) tasks were used as near transfer tasks. On each trial a fixation cross was presented for 750 ms, followed by a sequence of digits (0–9) displayed one at a time at a rate of 1000 ms in the center of the screen. At the end of the sequence, participants were asked to recall the digits in the same (DSF) or reverse (DSB) order that they appeared, typing their response into the answer box. There were two trials at each string length (DSF: 3–9; DSB: 2–8). If participants performed correctly on at least one trial of the two trials at a specific span length the string length was increased on the next trial. If participants performed incorrectly on both trials at a specific span length the task was terminated. The outcome measure was digit span, defined as the maximum string length at which participants repeated back the sequence in the correct order on 50% of trials.^7^ Further task details are reported in the Supplementary material.

#### 2.3.4. Far transfer task

A relational reasoning task modeled on Raven’s Progressive Matrices (Raven and Raven, 2003) investigated far transfer to abstract reasoning. A different set of stimuli were used at pre- and post-training. Trials increased in difficulty throughout the task. On each trial, a fixation cross was presented (500 ms) followed by a 3 × 3 grid of patterns with one pattern missing from the third row of the third column. Participants were required to click on the pattern according to logical rules that best completed the matrix from one of four patterns. Participants completed as many puzzles as possible within the 5-min time limit.^8^ The outcome measure was the number of correct solutions. Further task details and example trials are reported in the (Supplementary Figure 3).

#### 2.3.5. Acceptability questionnaire

Following the final post-training task participants were invited to complete an optional questionnaire that captured their thoughts on the training (Supplementary Figure 4). In this questionnaire, participants were asked (1) to rate how much they agreed with four statements relating to how challenging and engaging they found the training method, how likely they would be to adopt a method like this if it were offered in the future, and how optimal they thought the training schedule was for brain training, (2) what their motivation was for getting involved in the study, and (3) if there was anything they would change about the training.

### 2.4. Participant adherence and exclusions

Ninety participants were enrolled. Of these 90 participants, 11 did not begin the study. Seventy-one of the 79 participants who began the study completed the study (i.e., they completed the pre-training tasks, 16 training sessions, and post-training tasks), whilst eight did not (Figure 1). Three participants (two from the distributed-WMT group, and one from the intensive-WMT group) were excluded as they were unable to follow their assigned training schedule. Two participants were excluded due to a technical error during the training tasks (distributed-WMT: *n* = 1; intensive-active-control: *n* = 1). The final sample, therefore, comprised 67 participants [mean age: 66.12 years ± 6.51 (55–85 years); male/female: 17/50], which resulted in 12–18 participants per group in each statistical analysis. A more detailed summary of participant adherence and exclusions can be found in the Supplementary material.

### 2.5. Statistical analysis

Statistical analyses^9^ were conducted using R (Version 3.6.1) and IBM SPSS Statistics (Version 28). The alpha level was set to 0.05. If visual inspection of Q-Q plots showed that data were not normally distributed, non-parametric statistical tests were used. Extreme outliers were identified using Box and Whisker plots and defined as values that fell outside the 3rd quartile + 3*interquartile range and 1st quartile—3*interquartile range. Sensitivity analyses were run where extreme outliers were identified. Data were visualized in R using ggplot2 (Wickham, 2016) and the open-visualizations repository (van Langen, 2020).

#### 2.5.1. Demographics and baseline performance

To assess whether the randomization procedure led to comparable groups, groups were compared on demographics, pre-training transfer task performance, and S1 WMT performance using the appropriate statistical test (one-way ANOVA or chi-square test).

#### 2.5.2. WMT task effects^10^

First, we investigated training gain from the first session (S1) to the last session (S16) as a function of treatment (WMT/active-control) and schedule (distributed/intensive). For both the verbal and spatial n-back training tasks, we conducted a 2 × 2 × 2 analysis of variance (ANOVA) using mean n-back as the dependent variable, treatment and schedule as between-subjects factors, and session as a within-subjects factor.

Next, we investigated the dynamics of training task improvement in the WMT groups via two steps:

Step 1: We investigated how many training sessions were needed to observe a significant improvement relative to the first session, and whether this differed between training schedules. For this, we ran a 2 × 2 ANOVA, with mean n-back as the dependent variable, group (distributed-WMT/intensive-WMT) as a between-subjects factor, and session (S1/SN*) as a within-subjects factor (SN refers to each subsequent session after S1, i.e., 15 ANOVAs for each task). *P*-values for group, session, and group x session effects were corrected for multiple comparisons using a 5% false discovery rate [FDR; Benjamini and Hochberg (1995) procedure].

Step 2: We investigated whether the point of asymptotic-like performance differed between groups. For this, the data for each participant were first smoothed using a sliding window average to attenuate volatility in performance from session-to-session. Specifically, for each participant, performance at S1 + S2 was averaged, performance at S2 + S3 was averaged and so on, resulting in 15 data points (smallest smoothing unit possible). As learning effects were non-linear (see Supplementary Table 1), following Jaeggi et al. (2020) we then used spline regression to model two separate learning curves on the smoothed data. For each participant, learning rate was modeled using linear splines with one knot location (i.e., inflection point) at data point two (i.e., S2 + S3). Linear splines were specified iteratively through to data point 14 (i.e., at all data points except the first and last points). The R-squared value of each model was compared and the model with the highest R-squared was selected as the spline regression model for that participant. Two measures were compared between groups using Mann-Whitney U *t*-tests: the slope of the first segment (i.e., learning curve before asymptotic-like performance) and the data point number of the knot location (i.e., the point at which asymptotic-like performance was reached).

#### 2.5.3. Transfer task effects

For each of the transfer tasks, a 2 × 2 × 2 ANOVA was performed with treatment (WMT/active-control) and schedule (distributed/intensive) as between-subjects factors and session (pre-/post-training) as a within-subjects factor.

#### 2.5.4. Attrition and acceptability

A chi-square test compared attrition rate [(number that did not complete the intervention/number assigned) × 100] between groups. To explore responses to the questionnaire numerical values were assigned to each Likert scale response (1 = strongly disagree, 2 = disagree, 3 = neither agree nor disagree, 4 = agree, 5 = strongly agree). A 2 (treatment: WMT/active-control) × 2 (schedule: distributed/intensive) ANOVA was run for each Likert scale question.

## 3. Results

### 3.1. Demographics and baseline performance

There were no group differences in participant demographics, pre-training transfer task performance, or WMT task performance at the first session (Supplementary Table 4).

### 3.2. WMT effects

**FIGURE 4:**
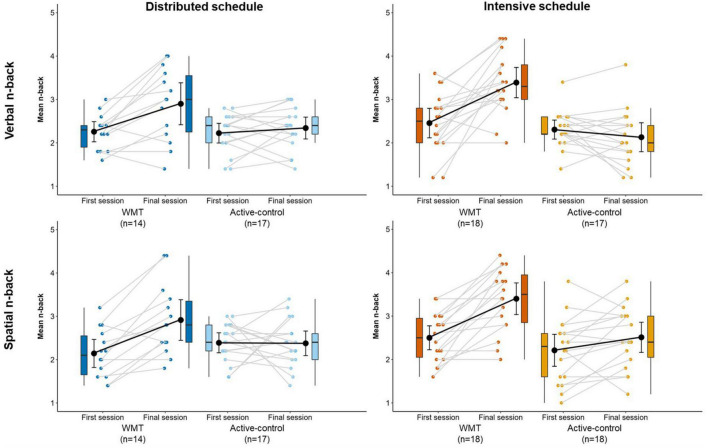
Performance on the verbal **(top)** and spatial **(bottom)** n-back training tasks at the first and final training sessions, separately for distributed groups **(left)** and intensive groups **(right)**. Box plots represent the median (crossbars), 1st and 3rd quartile (upper and lower hinges, respectively), and the range of the data (whiskers). Single data points represent individual participants. Black circles and error bars show the group mean and 95% confidence interval.

**FIGURE 5:**
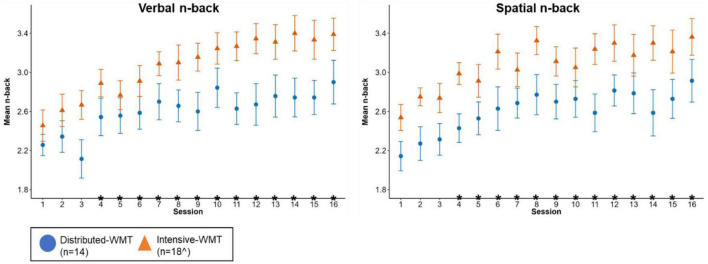
Verbal **(left)** and spatial **(right)** n-back task performance as a function of training schedule [error bars represent standard error of the mean; *represents a significant difference relative to S1 across groups (i.e., a session effect); ^for spatial n-back S1 vs. S4 and S1 vs. S7 had *n* = 17 in the intensive-WMT group due to excluded data for one participant at S4 and another participant at S7].

Figure 4 illustrates WMT task performance at the first (S1) and final (S16) sessions (Supplementary Table 5 contains descriptive statistics). Figure 5 displays WMT task performance at each session as a function of training schedule.

#### 3.2.1. Verbal n-back

There was a significant main effect of treatment, main effect of session, and treatment x session interaction (all *p* < 0.001; Table 2). Follow-up *t*-tests showed a significant difference between WMT and active-control groups at the final session [*t*_(64)_ = 5.55, *p* < 0.001, *g* = 1.35] but no difference at the first session [*t*_(64)_ = 0.84, *p* = 0.407, *g* = 0.20]. The WMT group showed significantly better performance at the final session relative to the first session [*t*_(31)_ = 5.75, *p* < 0.001, *g* = 0.99]; whereas active-controls did not [*t*_(33)_ = 0.33, *p* = 0.742, *g* = 0.06].

**TABLE 2:** WMT and transfer task performance: inferential statistics.

	ANOVA			
	***F***	***p***	**η_p_^2^**	**BF_10_**
**Verbal n-back**				
Treatment (WMT/active-control)	16.61	<0.001***	0.21	1299.05
Session (first/final)	21.70	<0.001***	0.26	15.01
Schedule (distributed/intensive)	1.29	0.260	0.02	0.41
Treatment × session	25.20	<0.001***	0.29	32.70
Session × schedule	0.00	0.991	0.00	0.20
Treatment × schedule	2.77	0.101	0.04	0.92
Treatment × session × schedule	3.22	0.078	0.05	0.58
**Spatial n-back**				
Treatment (WMT/active-control)	7.20	0.009*	0.10	16.67
Session (first/final)	36.98	<0.001***	0.37	177.42
Schedule (distributed/intensive)	2.13	0.149	0.03	0.61
Treatment × session	18.42	<0.001***	0.23	3.81
Session × schedule	1.87	0.177	0.03	0.39
Treatment × schedule	2.61	0.111	0.04	0.93
Treatment × session × schedule	0.32	0.572	0.01	0.18
**DSF**				
Treatment (WMT/active-control)	0.11	0.739	0.00	0.23
Session (pre-/post-training)	1.15	0.289	0.02	0.31
Schedule (distributed/intensive)	1.59	0.213	0.03	0.59
Treatment × session	0.77	0.385	0.01	0.22
Session × schedule	0.53	0.469	0.01	0.22
Treatment × schedule	0.01	0.908	0.00	0.20
Treatment × session × schedule	2.03	0.160	0.03	0.35
**DSB**				
Treatment (WMT/active-control)	0.11	0.737	0.00	0.23
Session (pre-/post-training)	1.51	0.224	0.03	0.26
Schedule (distributed/intensive)	0.32	0.572	0.01	0.23
Treatment × session	1.19	0.281	0.02	0.22
Session × schedule	0.06	0.812	0.00	0.19
Treatment × schedule	2.24	0.140	0.04	1.07
Treatment × session × schedule	3.33	0.073	0.05	0.31
**Relational reasoning**				
Treatment (WMT/active-control)	2.54	0.116	0.04	0.67
Session (pre-/post-training)	20.65	<0.001***	0.25	9.16
Schedule (distributed/intensive)	0.88	0.351	0.01	0.27
Treatment × session	3.45	0.068	0.05	0.40
Session × schedule	0.47	0.494	0.01	0.22
Treatment × schedule	3.89	0.053	0.06	1.62
Treatment × session × schedule	0.67	0.416	0.01	0.21

*p < 0.05, ***p < 0.001. BF_10_, Bayes factor, BF > 1 indicates evidence in favor of the alternative hypothesis.

#### 3.2.2. Spatial n-back

There was a significant main effect of treatment (*p* < 0.05), main effect of session (*p* < 0.001), and treatment x session interaction (*p* < 0.001; Table 2). Follow-up *t*-tests showed a significant difference between WMT and active-control groups at the final session [*t*_(65)_ = 4.26, *p* < 0.001, *g* = 1.03] but no difference at the first session [*t*_(65)_ = 0.32, *p* = 0.751, *g* = 0.08]. The WMT group showed significantly better performance at the final session relative to the first session [*t*_(31)_ = 7.86, *p* < 0.001, *g* = 1.36]; whereas active-controls did not [*t*_(34)_ = 1.26, *p* = 0.218, *g* = 0.21].

For both the verbal and spatial n-back tasks, performance at the first session was significantly worse than performance at the fourth session through to the last session across both WMT groups (i.e., a main effect of session). These *p*-values survived FDR correction. No group or group x session effects survived FDR correction (Supplementary Table 6).

Data for the first slope (spatial n-back) were not normally distributed for the distributed-WMT group. For consistency, non-parametric Mann-Whitney *U*-tests were run for each comparison in Table 3. There were no group differences in the value of the first slope or the knot location on the verbal or spatial n-back tasks. Findings remained the same after removing two extreme outliers (first slope) from the distributed-WMT group during the spatial n-back.

**TABLE 3:** Comparison of spline regression values.

	Distributed-WMT	Intensive-WMT	Mann-Whitney *U*-test			
	**M ± SD (median)**	**M ± SD (median)**	***U***	***p***	***g***	**BF_10_**
**Verbal n-back (*n*: DT = 14; IT = 18)**						
First slope	0.09 ± 0.09 (0.08)	0.10 ± 0.13 (0.11)	122.00	0.879	0.08	0.44
Knot location	8.50 ± 3.39 (8.50)	8.11 ± 3.64 (8.00)	117.00	0.731	0.11	0.38
**Spatial n-back (*n*: DT = 14; IT = 16)**						
First slope	0.17 ± 0.21 (0.14)	0.13 ± 0.12 (0.11)	103.00	0.708	0.29	0.34
Knot location	7.79 ± 4.10 (6.50)	8.50 ± 3.62 (9.50)	100.50	0.631	0.18	0.35

BF_10_, Bayes factor, BF > 1 indicates evidence in favor of the alternative hypothesis; DT, distributed-WMT; IT, intensive-WMT.

### 3.3. Near transfer effects

**FIGURE 6:**
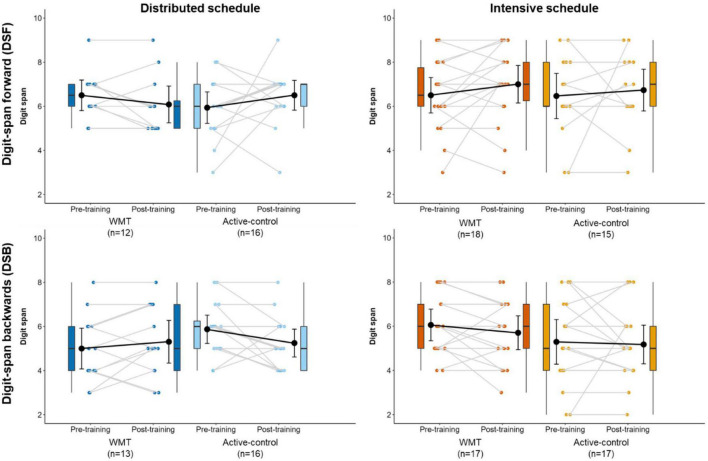
Performance on the DSF **(top)** and DSB **(bottom)** near transfer tasks at pre-training and post-training, separately for distributed groups **(left)** and intensive groups **(right)**. Box plots represent the median (crossbars), 1st and 3rd quartile (upper and lower hinges, respectively), and the range of the data (whiskers). Single data points represent individual participants. Black circles and error bars show the group mean and 95% confidence interval.

Figure 6 illustrates DSF and DSB performance at pre- and post-training (Supplementary Table 5 contains descriptive statistics). There were no significant main effects or interactions for the DSF and DSB (Table 2). The findings for the DSB task stayed the same after removing one participant who scored ceiling performance at pre- and post-training.

### 3.4. Far transfer effects

**FIGURE 7:**
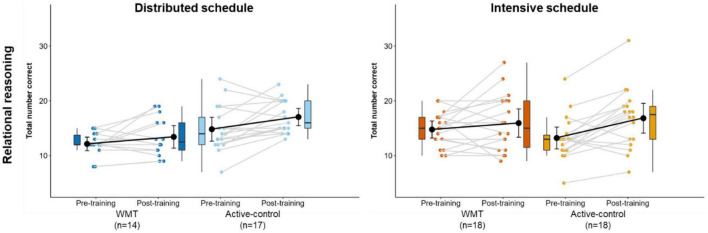
Performance on the relational reasoning task at pre-training and post-training, separately for distributed groups **(left)** and intensive groups **(right)**. Box plots represent the median (crossbars), 1st and 3rd quartile (upper and lower hinges, respectively), and the range of the data (whiskers). Single data points represent individual participants. Black circles and error bars show the group mean and 95% confidence interval.

Figure 7 illustrates relational reasoning performance at pre- and post-training (Supplementary Table 5 contains descriptive statistics). There was a significant main effect of session (*p* < 0.001), indicating significantly better performance at post-training relative to pre-training across the groups. There were no other significant main effects or interactions (Table 2). After removing one extreme outlier (intensive-active-control), the treatment x schedule interaction was significant (*p* = 0.011).

### 3.5. Attrition and acceptability

The overall attrition rate was 10.13% (started: *n* = 79; completed: *n* = 71). There was no significant difference in attrition rate between groups (χ2 = 12.00, *p* = 0.213; distributed-WMT: 15.00%; intensive-WMT: 14.29%; distributed-active-control: 0.00%; intensive-active-control: 10.00%; Figure 1). Moreover, there was no significant difference in participant demographics between participants who completed the study and participants who did not (Supplementary Table 7).

Of the 71 participants that completed the study, 93% completed the end-of-training questionnaire (*n* = 66; distributed-WMT: *n* = 15; intensive-WMT: *n* = 17; distributed-active-control: *n* = 16; intensive-WMT: *n* = 18). The majority of participants agreed or strongly agreed that the training was challenging (92%) and engaging (76%) and that they would adopt a cognitive intervention method like this if it were offered to them in the future (65%) (Supplementary Figure 6). There were no significant differences in responses to Likert questions depending on treatment or schedule (Supplementary Table 8).

## 4. Discussion

The objective of this study was to investigate the effect of training schedule on training and transfer task effects of WMT in healthy older adults. Participants completed sixteen WMT or active-control sessions over eight (distributed) or four (intensive) weeks. The participants successfully performed the cognitively demanding intervention using their own devices, online at home, and with minimal contact with the researcher. Consistent with our hypothesis, we observed an improvement in WMT task performance in the WMT group, but no change in the active-control group. However, contrary to our predictions, we found no evidence of transfer effects and the distribution of training sessions had no impact on WMT or transfer task effects.

### 4.1. WMT and transfer task effects

The magnitude of the training effect on the verbal and spatial WMT tasks was large. This is in keeping with previous meta-analyses of WMT in healthy older adults that report large effect sizes on trained tasks (Nguyen et al., 2019; Sala et al., 2019). For both WMT tasks, a significant improvement in performance relative to the first session was observed from session four onward. This is similar to an existing WMT study in healthy older adults that observed significant improvement in WMT task performance from session five (Bürki et al., 2014).

We observed greater performance at post-training relative to pre-training regardless of treatment or schedule allocation (i.e., a main effect of session) on the abstract relational reasoning task. The marginal (*p* = 0.068) treatment (WMT/active-control) x session (pre-/post-training) interaction for the relational reasoning task was driven by a steeper increase in performance in the active-control group relative to the WMT group. An increase in performance on this far transfer task was not expected for the active-control group. We might speculate that improvement in the active-control group arose from the similarity in structural properties of the visual search active-control task and the relational reasoning task. Specifically, both tasks required scanning a matrix and selecting an object from an array of choices.

We did not observe any significant transfer effects on a digit-span task (near transfer, measuring WM). This finding adds to the conflicting evidence base for near transfer effects following WMT. For instance, some studies report improved digit span following WMT in older adults (DSF: McAvinue et al., 2013; Heinzel et al., 2014), whilst others do not (DSF/DSB: Buschkuehl et al., 2008; Dahlin et al., 2008; Richmond et al., 2011; DSB: McAvinue et al., 2013; Heinzel et al., 2014).

An absence of near and far transfer effects is in contrast with Penner et al. (2012) findings. Using the same training schedules, they reported significantly greater near and far transfer effects in the distributed-WMT group (i.e., two sessions per week for 8 weeks) relative to controls. However, Penner et al. (2012) study used a no-contact control group as opposed to an active-control group like the present study, which might explain our different findings. Our observation of a session effect on the relational reasoning task highlights the importance of using an active-control group in WMT research. Overall, the absence of transfer effects is consistent with meta-analyses of WMT in older adults that have reported smaller or null effects of near and far transfer (Nguyen et al., 2019; Sala et al., 2019).

There are several possible explanations for the lack of transfer observed. For example, a lack of evidence for near transfer may stem from a reliance on rehearsal to perform the digit-span task (Buschkuehl et al., 2008). Rehearsal is a well-learned process that may be automatic and therefore difficult to modulate (Buschkuehl et al., 2008). Moreover, studies that have evidenced far transfer to other tests of fluid intelligence (e.g., abstract reasoning) have administered WMT tasks that place a high demand on executive functioning, such as task-switching (Karbach and Kray, 2009) and dual n-back paradigms (Jaeggi et al., 2008). We used a hybrid approach (i.e., an adaptive procedure with task diversification) to promote engagement and motivation (Borella et al., 2010). However, participants only trained on a single domain, verbal or spatial, at any one time. Therefore, the demand for executive control may not have been high enough to facilitate transfer to other domains of fluid intelligence (Karbach and Kray, 2009; Heinzel et al., 2014).

Finally, the adaptive feature of the n-back training task is believed to facilitate constant engagement of executive processes and subsequently dissuade the development of automatic processes and task-specific strategies (Jaeggi et al., 2008). However, a study directly testing the mechanism(s) of WMT transfer has suggested that n-back training results in the acquisition of a set of strategies, as opposed to domain-general mechanisms (e.g., strengthening of a process utilized by various tasks) (Linares et al., 2019). Specifically, Linares et al. (2019) observed transfer only to tasks with close structural similarity (known as nearest transfer), like tasks with information displayed in the same manner (e.g., in boxes) and requiring the same procedural steps (e.g., concentrating on the current stimulus to be remembered, retrieving the previous stimulus, and then modifying what is to be remembered). Therefore, the lack of structural similarity between the WMT and transfer tasks used in this study might play a role in the lack of transfer.

### 4.2. Training schedule

Similar training effects were observed irrespective of whether participants trained two times per week for 8 weeks (distributed-WMT) or four times a week for 4 weeks (intensive-WMT). We extended the restricted range of spacing schedules used by Jaeggi et al. (2020). Nevertheless, the lack of evidence for an impact of schedule intensity is consistent with Jaeggi et al. (2020) findings. Thus, it seems that the *spacing effect* observed during various other learning instances (e.g., language learning; Kim and Webb, 2022) might not apply in the context of WMT in healthy aging, owing to no obvious benefit of distributed practice. Ultimately, our findings suggest that comparable benefits could be observed when using less intensive schedules that may be more easily accommodated into a person’s life. As a next step, it would be informative to investigate whether less regular schedules (e.g., a combination of an intense training period followed by a distributed training period, or vice versa) or allowing individuals to choose their own training schedule would result in similar training benefits.

One speculative reason as to why a spacing effect is not observed on WMT in healthy aging but is a common observation in other learning instances could reflect a difference between WM and long-term memory. More specifically, WMT could be viewed as training a skill, whilst learning a language, for instance, requires encoded material to be remembered. These two processes may be differentially affected by spacing. Nevertheless, this speculation does not explain why some studies investigating the spacing effect in other populations (e.g., healthy younger adults and children) have reported a benefit of spaced over massed training (Penner et al., 2012; Wang et al., 2014).

### 4.3. Feasibility

Most of the participants were successfully able to perform the demanding intervention using their own devices, online at home, and with minimal contact with the researcher. Beyond simply completing the intervention, they demonstrated significant improvements in WMT task performance, consistent with other lab-based studies (Nguyen et al., 2019). Additionally, the majority of participants agreed that the WMT was challenging and engaging and that they would adopt a cognitive intervention like this if it were offered to them in the future. This success is noteworthy as if WMT is to have a widespread reach it is essential that it can be administered outside the lab in an unsupervised environment without the need for externally supplied equipment.

### 4.4. Study limitations

We should consider the contribution of this study in light of several limitations. Firstly, despite the accessibility of home-based interventions, we must acknowledge that relative to lab-based interventions, unsupervised research, performed outside the lab, using participants’ devices, has reduced experimental control. For instance, there was no researcher present to clarify instructions or assist with technical problems in real time. To attenuate potential problems arising from reduced control several methods were implemented to ensure the quality of the training provided. For example, to enable understanding of the task requirements, participants could re-visit task instructions and repeat practice trials as many times as needed. Additionally, lack of engagement and/or understanding of the tasks (e.g., letting the task run through without responding, scoring zero correct responses) and instances of technical error (e.g., crashing and re-starting) were identified during data analyses. We believe that these methodological choices have allowed us to ensure the quality of the training sufficiently whilst conducting remote research.

Secondly, our conclusions are only applicable to the WMT tasks used in this study, so we cannot conclude whether the spacing effect would be observed using other WMT paradigms. Next, the absence of a follow-up assessment prohibits an investigation of whether improvements on the training tasks persist over time and whether spacing effects arise after a longer delay (Cepeda et al., 2006). Moreover, it is desirable within WMT research that the only difference between the WMT task and the active-control task is the demand on WM (Simons et al., 2016; Gobet and Sala, 2022). Although the active-control tasks were designed to mirror the verbal and spatial elements of the WMT, future research should implement *adaptive* active-control tasks to further isolate the effect of WMT (Simons et al., 2016; Gobet and Sala, 2022).

Furthermore, a higher percentage of participants reported having tertiary education (73%, *n* = 49) compared to the UK population (39% of 55–64-year-olds; OECD, 2022). Therefore, the sample might not be representative of the typical healthy older population, reducing the generalizability of our findings. Moreover, the sample (mean age: 66 years) can be considered a young-old sample (Borella et al., 2010). Thus, future studies should investigate spacing effects in older-old adults (85 + years).

Finally, the intervention was not double-blinded. However, the absence of face-to-face interaction and minimal participant-researcher email correspondence reduced the likelihood of unintentional differences in researcher behavior dependent on group allocation. Moreover, the active-control task had some degree of face validity as a cognitive training intervention. For instance, given the commercialized nature of brain training we believe that the wordsearch and visual search control tasks would have been perceived as an *active* intervention by the participants, providing good face validity (Green et al., 2019). Additionally, the study advertisements stated that the study aimed to compare two training schedules, without reference to the WMT and active-control group allocation. This conscious effort to create equivalent expectations in the WMT and active-control groups served to promote participant blinding to treatment allocation.

## 5. Conclusion

The current study investigated the effect of training schedule on training and transfer effects of WMT in healthy older adults. The magnitude of the training effect on the verbal and spatial WMT tasks was large. However, we observed no evidence of near and far transfer measures of WM or abstract relational reasoning. Despite the absence of transfer effects, this study suggests that healthy older adults can successfully complete a cognitively demanding intervention unsupervised using their personal devices at home. Our findings suggest that similar effects of WMT can be observed in healthy older adults irrespective of the training schedule, at least using the two schedules tested here, suggesting that intensive training is just as effective as distributed training.

## Data availability statement

The datasets analyzed in this study can be found in the figshare data repository (https://doi.org/10.48420/22071683).

## Ethics statement

The studies involving human participants were reviewed and approved by the University of Manchester Research Ethics Committee (Ref: 2020-8907-16162). The patients/participants provided their written informed consent to participate in this study.

## Author contributions

SB responsible for study conception, methodology, participant recruitment, data collection, data wrangling, statistical analysis, data visualization, writing the original draft, and editing subsequent drafts. LB, JT, and GP guided study conception, data collection, and statistical analysis, and reviewed manuscript drafts. All authors contributed to the article and approved the submitted version.

## Footnotes

1
http://www.gpower.hhu.de/

2 Participants were allocated on an alternating basis until group sizes needed to be balanced due to drop out.

3
https://gorilla.sc/

4 Based on the adaptive n-back available here: https://app.gorilla.sc/openmaterials/53268.

5 The adaptive n-back training task was programmed up to 5-back. There was no evidence of ceiling performance as no participant consistently reached 100% performance at 5-back (one participant reached 100% performance at 5-back on one block during one session of the spatial n-back).

6 There was no evidence of ceiling effects on the visual search task as no participant answered all available trials during any session.

7 On the DSF two participants scored the maximum number of correct trials at pre-training and one participant at post-training. No participants scored the maximum at both pre- and post-training. On the DSB three participants scored the maximum number of correct trials at pre-training and two at post-training (one participant achieved maximum at both pre- and post-training–findings stayed the same when removing this participant from the analysis).

8 There was no evidence of ceiling effects on the far transfer task as no participants achieved the maximum score of 40 correct trials at pre- or post-training.

9 Spline regression and Bayes Factor analyses were performed in R. All other statistical analyses were performed in SPSS.

10 Complementary analyses performed on smoothed data (using a sliding window average to attenuate volatility) are reported in the (Supplementary Figure 5 and Supplementary Tables 2, 3) [note: conclusions are the same as the analyses on the raw (unsmoothed) data].

## References

[B1] Anguera J. A. Boccanfuso J. Rintoul J. L. Al-Hashimi O. Faraji F. Janowich J. (2013). Video game training enhances cognitive control in older adults. *Nature* 501 97–101. 10.1038/nature12486 24005416 PMC3983066

[B2] Atkinson A. L. Baddeley A. D. Allen R. J. (2018). Remember some or remember all? Aging and strategy effects in visual working memory. *Q. J. Exp. Psychol.* 71 1561–1573. 10.1080/17470218.2017.1341537 28812424

[B3] Baddeley A. D. Hitch G. (1974). “Working Memory,” in *The psychology of learning and motivation*, ed. Bower G. H. (Cambridge, MA: Academic Press), 47–89. 10.1016/S0079-7421(08)60452-1

[B4] Benjamini Y. Hochberg Y. (1995). Controlling the false discovery rate: A practical and powerful approach to multiple testing. *J. R. Stat. Soc.* 57 289–300. 10.1111/j.2517-6161.1995.tb02031.x

[B5] Borella E. Carretti B. Riboldi F. De Beni R. (2010). Working memory training in older adults: Evidence of transfer and maintenance effects. *Psychol. Aging* 25 767–778. 10.1037/a0020683 20973604

[B6] Brehmer Y. Shing Y. L. Heekeren H. R. Lindenberger U. Bäckman L. (2016). Training-induced changes in subsequent-memory effects: No major differences among children, younger adults, and older adults. *NeuroImage* 131 214–225. 10.1016/j.neuroimage.2015.11.074 26673112

[B7] Brockmole J. R. Logie R. H. (2013). Age-related change in visual working memory: A study of 55,753 participants aged 8–75. *Front. Psychol.* 4:12. 10.3389/fpsyg.2013.00012 23372556 PMC3557412

[B8] Bürki C. N. Ludwig C. Chicherio C. de Ribaupierre A. (2014). Individual differences in cognitive plasticity: An investigation of training curves in younger and older adults. *Psychol. Res.* 78 821–835. 10.1007/s00426-014-0559-3 24652343

[B9] Buschkuehl M. Jaeggi S. M. Hutchison S. Perrig-Chiello P. Däpp C. Müller M. (2008). Impact of working memory training on memory performance in old-old adults. *Psychol. Aging* 23 743–753. 10.1037/a0014342 19140646

[B10] Cepeda N. J. Pashler H. Vul E. Wixted J. T. Rohrer D. (2006). Distributed practice in verbal recall tasks: A review and quantitative synthesis. *Psychol. Bull.* 132 354–380. 10.1037/0033-2909.132.3.354 16719566

[B11] Chai W. J. Abd Hamid A. I. Abdullah J. M. (2018). Working memory from the psychological and neurosciences perspectives: A review. *Front. Psychol.* 9:401. 10.3389/fpsyg.2018.00401 29636715 PMC5881171

[B12] Cowan N. (2014). Working memory underpins cognitive development, learning, and education. *Educ. Psychol. Rev.* 26 197–223. 10.1007/s10648-013-9246-y 25346585 PMC4207727

[B13] Dahlin E. Nyberg L. Bäckman L. Neely A. S. (2008). Plasticity of executive functioning in young and older adults: Immediate training gains, transfer, and long-term maintenance. *Psychol. Aging* 23 720–730. 10.1037/a0014296 19140643

[B14] Del Missier F. Mäntylä T. Hansson P. Bruine de Bruin W. Parker A. M. Nilsson L. G. (2013). The multifold relationship between memory and decision making: An individual-differences study. *J. Exp. Psychol.* 39 1344–1364. 10.1037/a0032379 23565790 PMC4160880

[B15] Diamond A. (2013). Executive functions. *Annu. Rev. Psychol.* 64 135–168. 10.1146/annurev-psych-113011-143750 23020641 PMC4084861

[B16] Duda B. M. Sweet L. H. (2019). Functional brain changes associated with cognitive training in healthy older adults: A preliminary ALE meta-analysis. *Brain Imaging Behav.* 14 1247–1262. 10.31234/osf.io/5kj3z 30900077

[B17] Fournet N. Roulin J. L. Vallet F. Beaudoin M. Agrigoroaei S. Paignon A. (2012). Evaluating short-term and working memory in older adults: French normative data. *Aging Ment. Health* 16 922–930. 10.1080/13607863.2012.674487 22533476

[B18] Garg S. Taylor J. R. Booth S. J. Pye E. Green J. Vassallo G. Jung J. Hulleman J. Pobric G. (2022). A pilot study of cognitive training combined with non-invasive brain stimulation in neurofibromatosis type 1. *Transl. Psychiatry*, (under review)

[B19] Gobet F. Sala G. (2022). Cognitive training: A field in search of a phenomenon. *Perspect. Psychol. Sci.* 18 125–141. 10.1177/17456916221091830 35939827 PMC9903001

[B20] Green C. S. Bavelier D. Kramer A. F. Vinogradov S. Ansorge U. Ball K. K. (2019). Improving methodological standards in behavioral interventions for cognitive enhancement. *J. Cogn. Enhanc.* 3 2–29. 10.1007/s41465-018-0115-y

[B21] Heinzel S. Schulte S. Onken J. Duong Q. L. Riemer T. G. Heinz A. (2014). Working memory training improvements and gains in non-trained cognitive tasks in young and older adults. *Aging Neuropsychol. Cogn.* 21 146–173. 10.1080/13825585.2013.790338 23639070

[B22] Hou J. Jiang T. Fu J. Su B. Wu H. Sun R. (2020). The long-term efficacy of working memory training in healthy older adults: A systematic review and meta-analysis of 22 randomized controlled trials. *J. Gerontol.* 75 e174–e188. 10.1093/geronb/gbaa077 32507890

[B23] Jaeggi S. M. Buschkuehl M. Jonides J. Perrig W. J. (2008). Improving fluid intelligence with training on working memory. *Proc. Natl. Acad. Sci.* 105 6829–6833. 10.1073/pnas.0801268105 18443283 PMC2383929

[B24] Jaeggi S. M. Buschkuehl M. Parlett-Pelleriti C. M. Moon S. M. Evans M. Kritzmacher A. (2020). Investigating the effects of spacing on working memory training outcome: A randomized, controlled, multisite trial in older adults. *J. Gerontol.* 75 1181–1192. 10.1093/geronb/gbz090 31353413 PMC7265810

[B25] Jaroslawska A. J. Gathercole S. E. Allen R. J. Holmes J. (2016). Following instructions from working memory: Why does action at encoding and recall help? *Mem. Cogn.* 44 1183–1191. 10.3758/s13421-016-0636-5 27443320 PMC5085979

[B26] Karbach J. Kray J. (2009). How useful is executive control training? Age differences in near and far transfer of task-switching training. *Dev. Sci.* 12 978–990. 10.1111/j.1467-7687.2009.00846.x 19840052

[B27] Karbach J. Verhaeghen P. (2014). Making working memory work: A meta-analysis of executive-control and working memory training in older adults. *Psychol. Sci.* 25 2027–2037. 10.1177/0956797614548725 25298292 PMC4381540

[B28] Kim S. K. Webb S. (2022). The effects of spaced practice on second language learning: A meta-analysis. *Lang. Learn.* 72 269–319. 10.1111/lang.12479

[B29] Klingberg T. Forssberg H. Westerberg H. (2002). Training of working memory in children with ADHD. *J. Clin. Exp. Neuropsychol.* 24 781–791. 10.1076/jcen.24.6.781.8395 12424652

[B30] Li Q. Long Q. Hu N. Tang Y. Chen A. (2020). N-back task training helps to improve post-error performance. *Front. Psychol.* 11:370. 10.3389/fpsyg.2020.00370 32218757 PMC7078347

[B31] Linares R. Borella E. Lechuga M. T. Carretti B. Pelegrina S. (2019). Nearest transfer effects of working memory training: A comparison of two programs focused on working memory updating. *PLoS One* 14:e0211321. 10.1371/journal.pone.0211321 30759135 PMC6373913

[B32] Lövdén M. Bäckman L. Lindenberger U. Schaefer S. Schmiedek F. (2010). A theoretical framework for the study of adult cognitive plasticity. *Psychol. Bull.* 136 659–676. 10.1037/a0020080 20565172

[B33] Matysiak O. Kroemeke A. Brzezicka A. (2019). Working memory capacity as a predictor of cognitive training efficacy in the elderly population. *Front. Aging Neurosci* 11:126. 10.3389/fnagi.2019.00126 31214015 PMC6554703

[B34] McAvinue L. P. Golemme M. Castorina M. Tatti E. Pigni F. M. Salomone S. (2013). An evaluation of a working memory training scheme in older adults. *Front. Aging Neurosci.* 5:20. 10.3389/fnagi.2013.00020 23734126 PMC3662021

[B35] Mewborn C. M. Lindbergh C. A. Stephen Miller L. (2017). Cognitive interventions for cognitively healthy, mildly impaired, and mixed samples of older adults: A systematic review and meta-analysis of randomized-controlled trials. *Neuropsychol. Rev.* 27 403–439. 10.1007/s11065-017-9350-8 28726168

[B36] Miyake A. Friedman N. P. Emerson M. J. Witzki A. H. Howerter A. Wager T. D. (2000). The unity and diversity of executive functions and their contributions to complex “frontal lobe” tasks: A latent variable analysis. *Cogn. Psychol.* 41 49–100. 10.1006/cogp.1999.0734 10945922

[B37] Nee D. E. Brown J. W. Askren M. K. Berman M. G. Demiralp E. Krawitz A. (2013). A meta-analysis of executive components of working memory. *Cereb. Cortex* 23 264–282. 10.1093/cercor/bhs007 22314046 PMC3584956

[B38] Nguyen L. Murphy K. Andrews G. (2019). Immediate and long-term efficacy of executive functions cognitive training in older adults: A systematic review and meta-analysis. *Psychol. Bull.* 145 698–733. 10.1037/bul0000196 30998045

[B39] OECD (2022). *Population with tertiary education.* Paris: OECD.

[B40] Penner I. K. Vogt A. Stöcklin M. Gschwind L. Opwis K. Calabrese P. (2012). Computerised working memory training in healthy adults: A comparison of two different training schedules. *Neuropsychol. Rehabil.* 22 716–733. 10.1080/09602011.2012.686883 22671966

[B41] Plassman B. L. Williams J. W. Jr. Burke J. R. Holsinger T. Benjamin S. (2010). Systematic review: Factors associated with risk for and possible prevention of cognitive decline in later life. *Ann. Intern. Med.* 153 182–193. 10.7326/0003-4819-153-3-201008030-00258 20547887

[B42] Prehn K. Flöel A. (2015). Potentials and limits to enhance cognitive functions in healthy and pathological ageing by tDCS. *Front. Cell. Neurosci*. 9:355. 10.3389/fncel.2015.00355 26441526 PMC4568338

[B43] Raven J. Raven J. (2003). “Raven progressive matrices,” in *Handbook of Nonverbal Assessment*, ed. McCallum R. S. (New York, NY: Plenum Publishers), 223–237. 10.1007/978-1-4615-0153-4_11

[B44] Reinhart R. M. G. Nguyen J. A. (2019). Working memory revived in older adults by synchronizing rhythmic brain circuits. *Nat. Neurosci.* 22 820–827. 10.1038/s41593-019-0371-x 30962628 PMC6486414

[B45] Richmond L. L. Morrison A. B. Chein J. M. Olson I. R. (2011). Working memory training and transfer in older adults. *Psychol. Aging* 26 813–822. 10.1037/a0023631 21707176

[B46] Sabia S. Singh-Manoux A. Hagger-Johnson G. Cambois E. Brunner E. J. Kivimaki M. (2012). Influence of individual and combined healthy behaviors on successful aging. *Can. Med. Assoc. J.* 184 1985–1992. 10.1503/cmaj.121080 23091184 PMC3519184

[B47] Sala G. Aksayli N. D. Tatlidil K. S. Gondo Y. Gobet F. (2019). Working memory training does not enhance older adults’ cognitive skills: A comprehensive meta-analysis. *Intelligence* 77:101386. 10.1016/j.intell.2019.101386

[B48] Shipstead Z. Redick T. S. Engle R. W. (2012). Is working memory training effective? *Psychol. Bull.* 138 628–654. 10.1037/a0027473 22409508

[B49] Simons D. J. Boot W. R. Charness N. Gathercole S. E. Chabris C. F. Hambrick D. Z. (2016). Do “brain-training” programs work? *Psychol. Sci. Public Interest* 17 103–186. 10.1177/1529100616661983 27697851

[B50] Soveri A. Antfolk J. Karlsson L. Salo B. Laine M. (2017). Working memory training revisited: A multi-level meta-analysis of n-back training studies. *Psychon Bull Rev.* 24 1077–1096. 10.3758/s13423-016-1217-0 28116702

[B51] Sweet L. H. (2011). “N-back paradigm,” in *Encyclopaedia of Clinical Neuropsychology*, eds Kreutzer J. S. DeLuca J. Caplan B. (Berlin: Springer), 1718–1719. 10.1007/978-0-387-79948-3_1315

[B52] Takeuchi H. Taki Y. Kawashima R. (2010). Effects of working memory training on cognitive functions and neural systems. *Rev. Neurosci.* 21 427–450. 10.1515/revneuro.2010.21.6.427 21438192

[B53] van Langen J. (2020). *Open-visualizations in R and python.* Available online at: https://github.com/jorvlan/open-visualizations (accessed August 5, 2022).

[B54] Wang Z. Zhou R. Shah P. (2014). Spaced cognitive training promotes training transfer. *Front. Hum. Neurosci.* 8:217. 10.3389/fnhum.2014.00217 24782744 PMC3989588

[B55] Wickham H. (2016). *ggplot2: Elegant graphics for data analysis.* Available online at: https://ggplot2.tidyverse.org (accessed August 5, 2022).

[B56] Wilson R. S. Beckett L. A. Barnes L. L. Schneider J. A. Bach J. Evans D. A. (2002). Individual differences in rates of change in cognitive abilities of older persons. *Psychol. Aging* 17:179. 10.1037/0882-7974.17.2.179 12061405

[B57] Wilson R. S. Boyle P. A. Segawa E. Yu L. Begeny C. T. Anagnos S. E. (2013). The influence of cognitive decline on well-being in old age. *Psychol. Aging* 28 304–313. 10.1037/a0031196 23421323 PMC3692607

[B58] Wischnewski M. Mantell K. E. Opitz A. (2021). Identifying regions in prefrontal cortex related to working memory improvement: A novel meta-analytic method using electric field modelling. *Neurosci. Biobehav. Rev.* 130 147–161. 10.1016/j.neubiorev.2021.08.017 34418436 PMC8511213

[B59] Zaninotto P. Batty G. D. Allerhand M. Deary I. J. (2018). Cognitive function trajectories and their determinants in older people: 8 years of follow-up in the English longitudinal study of aging. *J. Epidemiol. Community Health* 72 685–694. 10.1136/jech-2017-210116 29691286 PMC6204948

