# Supplementary material for: Experimental investigation of training schedule on home-based working memory training in healthy older adults
Source: Front Psychol. 2023 Apr 28;14:1165275. doi: 10.3389/fpsyg.2023.1165275 (PMC10175577; doi:10.3389/fpsyg.2023.1165275)
Supplement: Supplementary file 1 [file Data_Sheet_1.pdf]

Supplementary Material

Experimental investigation of training schedule on home-based working memory training in healthy older adults

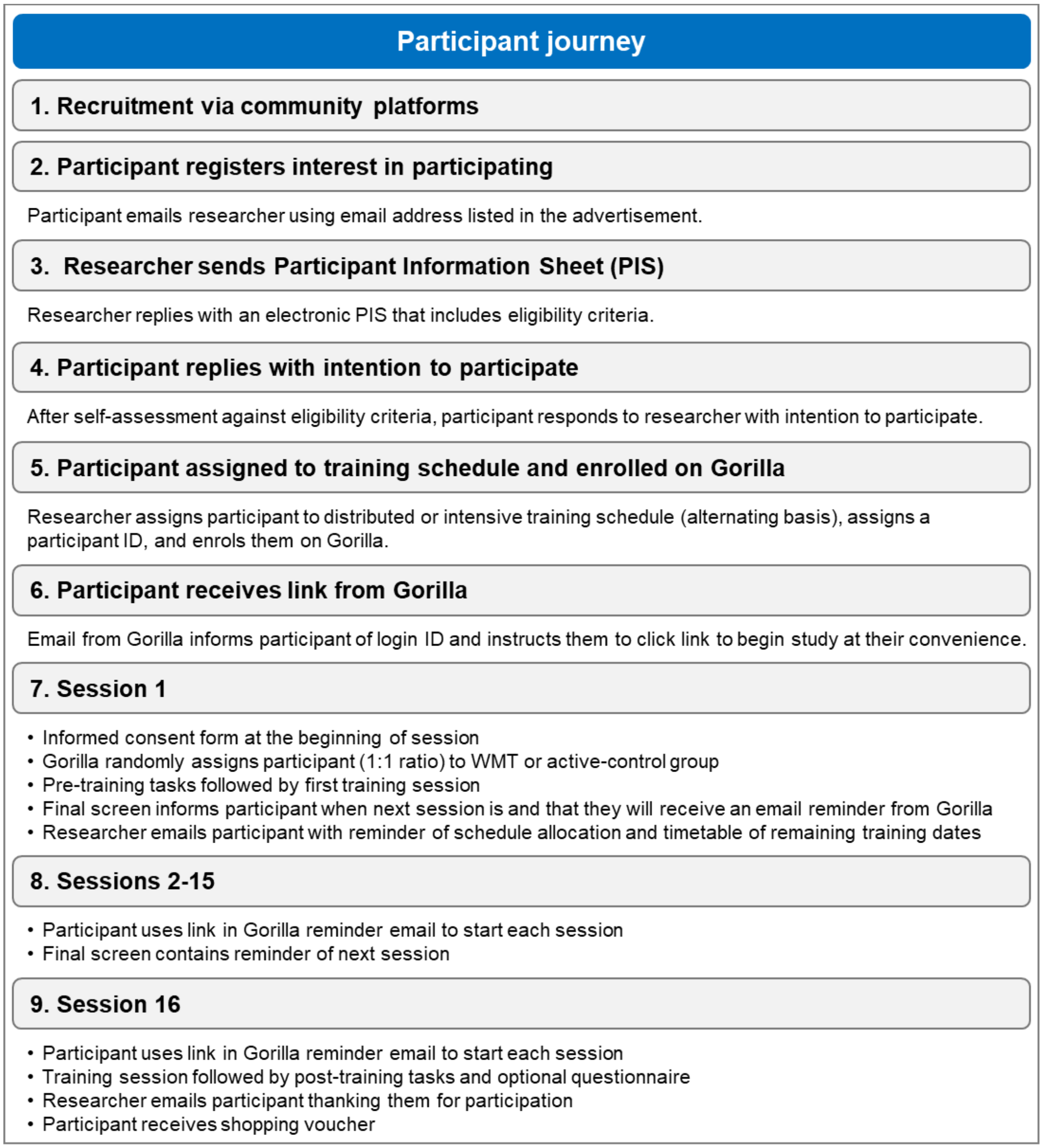

Figure S1. Participant journey.

(a) Distributed training schedule starting on a Tuesday

Week	Mon	Tue	Wed	Thu	Fri	Sat	Sun
1	/	S1	/	S2	/	/	/
2	/	S3	/	S4	/	/	/
3	/	S5	/	S6	/	/	/
4	/	S7	/	S8	/	/	/
5	/	S9	/	S10	/	/	/
6	/	S11	/	S12	/	/	/
7	/	S13	/	S14	/	/	/
8	/	S15	/	S16	/	/	/

(b) Intensive training schedule starting on a Tuesday

Week	Mon	Tue	Wed	Thu	Fri	Sat	Sun
1	/	S1	S2	S3	S4	/	/
2	/	S5	S6	S7	S8	/	/
3	/	S9	S10	S11	S12	/	/
4	/	S13	S14	S15	S16	/	/

Abbreviations: 'S' stands for session; / denotes no training.

Figure S2. Illustration of an example training schedule with the first session starting on a Tuesday. The **(a)** distributed group trained twice per week for eight weeks. The two sessions per week were spaced two days apart, with a five-day gap before the next week's training. The **(b)** intensive group trained four times per week for four weeks. The four sessions per week were performed on consecutive days, with a four-day gap before the next week's training.

Active-control tasks

Verbal (wordsearch, ~10 minutes) and spatial (visual search, ~10 minutes) control tasks were presented at each active-control session. These tasks were designed to mirror the verbal and spatial WMT tasks. They acted purely as an active-control and performance was not measured. However, participant engagement and understanding were monitored (see participant adherence and exclusions section).

The wordsearch task had four rounds in each session. In each round, a novel wordsearch (sourced from thewordsearch.com) was presented on screen. Participants had two minutes to locate and mark as many words as possible. There was a 15s instruction reminder before each round and a 60s break after two rounds. In each trial of the visual search task, a 4x4 array of objects was presented with a target object presented at the top of the screen. Participants were tasked with locating and clicking on the target object from the array. A different 4x4 array and target object was presented at each trial. There was a 60s break after five minutes. Images were sourced from the THINGS database (Hebart et al., 2019) and an Image Set made available by Long, Yu, and Konkle (2018).

Near transfer tasks

Computerized digit-span forward (DSF) and backward (DSB) tasks, well-known tests of working memory, were used as near transfer tasks. We used a modified version of the digit-span task described by Borella et al. (2013). On each trial a fixation cross was presented for 750ms, followed by a sequence of digits (0-9) displayed one at a time at a rate of 1000ms in the centre of the screen. At the end of the sequence, participants were asked to recall the digits in the same (DSF) or reverse (DSB) order that they appeared, typing their response into the answer box using their keyboard and pressing enter on completion. There was no time limit for a response. The digits presented in each sequence were a randomized selection of 0-9, where the first digit could not be a ‘0’ and the digit presented could not be the same as the one that followed it.

Each task started with a practice trial with a two-digit string length. Accuracy feedback was provided during the practice trial but not during the main task. Participants could re-visit the task instructions and repeat the practice trial before moving on to the main task. During the main task, there were two trials at each string length. In the DSF, the series started with three digits and continued to nine digits. In the DSB, the series started with two digits and continued to eight digits. If participants performed correctly on at least one trial of the two trials at a specific span length the string length was increased on the next trial. If participants performed incorrectly on both trials at a specific span length the task was terminated. The outcome measure was digit span, defined as the maximum string length at which participants repeated back the sequence in the correct order on 50% of trials.

Far transfer task

A relational reasoning task modelled on Raven’s Progressive Matrices (Raven & Raven, 2003) investigated far transfer to abstract reasoning. This task was chosen as visual analogy tasks are considered non-verbal tests of fluid intelligence (Bilker et al., 2015) and performance on relational reasoning tasks decline with age (Aichele et al., 2018, 2021). Stimuli were sourced from the matrix reasoning item bank (Chierchia et al., 2019). A different set of stimuli were used at pre- and post-training. Trials increased in difficulty throughout the task. According to Chierchia and colleagues (2019) criteria, ‘easy’ trials (trials

1-5) comprised of one-relational change (e.g., color), ‘medium’ trials (trials 6-21) comprised of two-relational changes (e.g., color/shape), and ‘hard’ trials (trials 22-40) comprised of three-relational changes (e.g., shape/color/position). Trials were in the same order for all participants. On each trial, a fixation cross was presented (500ms) followed by a 3x3 grid of patterns with one pattern missing from the third row of the third column. Participants were required to click on the pattern according to logical rules that best completed the matrix from one of four patterns. They were instructed to respond as quickly and as accurately as possible. The task started with two practice trials (one ‘easy’ and one ‘medium’ difficulty) with accuracy feedback provided. Participants could re-visit the task instructions and repeat the practice trials before moving onto the main task. Participants completed as many puzzles as possible within the five-minute time limit. The outcome measure was the number of correct solutions.

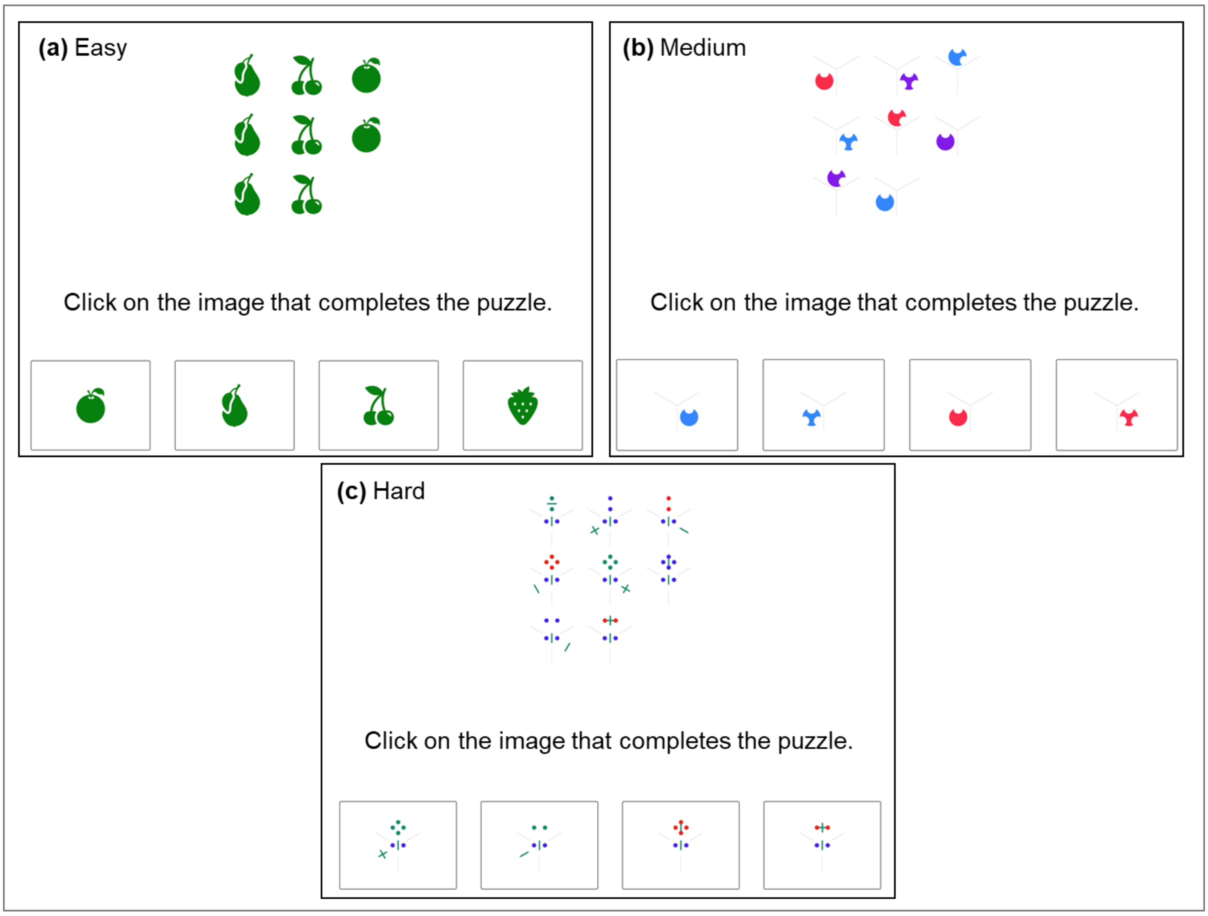

Figure S3. Example difficulty level (a) easy, (b) medium, and (c) hard for the relational reasoning task.

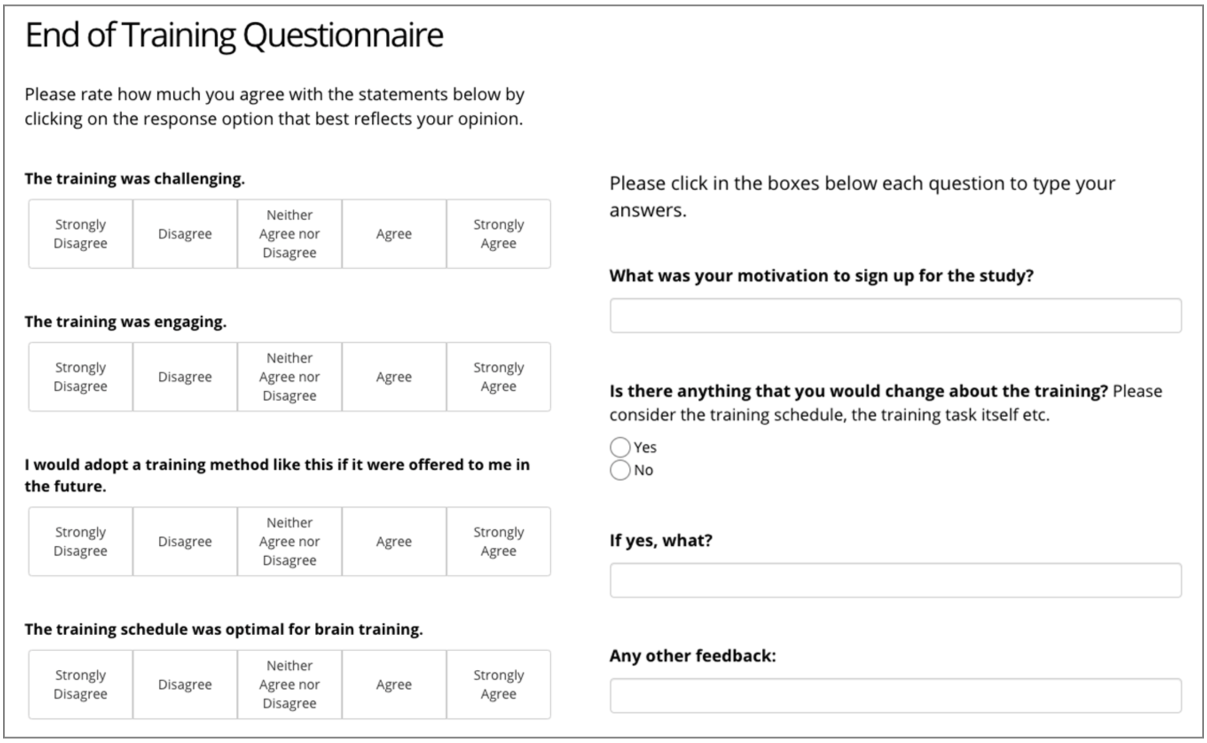

End of Training Questionnaire

Please rate how much you agree with the statements below by clicking on the response option that best reflects your opinion.

The training was challenging.

Strongly Disagree	Disagree	Neither Agree nor Disagree	Agree	Strongly Agree

The training was engaging.

Strongly Disagree	Disagree	Neither Agree nor Disagree	Agree	Strongly Agree

I would adopt a training method like this if it were offered to me in the future.

Strongly Disagree	Disagree	Neither Agree nor Disagree	Agree	Strongly Agree

The training schedule was optimal for brain training.

Strongly Disagree	Disagree	Neither Agree nor Disagree	Agree	Strongly Agree

Please click in the boxes below each question to type your answers.

What was your motivation to sign up for the study?

Is there anything that you would change about the training? Please consider the training schedule, the training task itself etc.

- Yes
- No

If yes, what?

Any other feedback:

Figure S4. Optional end-of-training questionnaire.

Participant adherence and exclusions

Ninety participants were enrolled on Gorilla. Of these 90 participants, 11 did not begin the study (n=3 experienced a technical obstacle to participation, e.g., only having access to an iPad when a laptop/desktop computer was required; n=8 reason unknown).

Seventy-one of the 79 participants who began the study completed the study (i.e., they completed the pre-training tasks, 16 training sessions, and the post-training tasks), whilst eight did not complete the study. Of the eight participants who did not complete the study: two withdrew due to health problems unrelated to the study (completed eight (intensive-active-control: n=1) and 15 sessions (distributed-WMT: n=1), respectively), one withdrew stating that the schedule was too demanding of their time (completed six sessions; intensive-active-control: n=1), three withdrew stating they were not enjoying the study (intensive-WMT: n=1 completed the pre-training tasks and withdrew partway through the first training task as they did not enjoy playing number games; distributed-WMT: n=1 withdrew during the pre-training tasks as they were bored and their concentration wandered; distributed-WMT: n=1 withdrew during S6 as they were finding the training frustrating), and two participants were requested by the researcher to discontinue their participation due to a lack of adherence[1] with the study protocol (intensive-WMT: n=1 did not engage with the pre-training tasks and training tasks during S1 and n=1 did not engage with the training tasks during S1, both participants let the tasks run through without engaging).

Participants were required to have fulfilled the training requirements to be eligible for inclusion in the statistical analyses. Specifically, they needed to have adhered to their allocated training schedule (i.e., distributed: two sessions per week for eight weeks; intensive: four sessions per week for four weeks) and completed 16 training sessions with evidence of engagement and understanding task requirements throughout (see below). Three participants were excluded as they were unable to follow their assigned training schedule (distributed-WMT: n=2, for one participant there was a 23-day gap between S5+S6, and for the other participant there was a 13-day gap between S14+S15; distributed-active-control: n=1, there was a 12-day gap between S2+S3).

At each training session, we checked for instances of technical error on the WMT, active-control, and transfer (where applicable) tasks. Due to technical error, only four of the eight verbal n-back blocks ran for one participant in the distributed-WMT group during S16. Thus, they did not fulfil the training requirements as they did not train for 16 complete training sessions and were therefore excluded from the analysis. Additionally, for one participant in the intensive-active-control group, the verbal n-back task re-started partway through S1. This resulted in the participant performing more than the required eight blocks, and although these blocks were adaptive in difficulty level, they did not follow the strict adaptive nature specified in section 2.3.1 of the main manuscript. As the data from this session was not comparable to the other participants' data it could not be used. Therefore, this participant was excluded from the verbal n-back analysis. For two other participants (n=2: intensive-WMT) the WMT task re-started partway through the spatial n-back task during S4 and S7, respectively. However, this was still considered as meeting the training requirements and did

[1] Each participant's performance was checked during their first session to ensure that they were engaging with the study. For participants in the active-control groups engagement with the wordsearch task was checked after each session. For the WMT and visual search tasks, thorough engagement checks were run following data collection (discussed in the remainder of this section).

not occur during the first or last session. There were no technical errors during the visual search and wordsearch control tasks or the transfer tasks.

Finally, at each session, we checked for evidence of a lack of engagement and/or understanding of task requirements in the WMT, active-control, and transfer (where applicable) tasks. Specifically, we checked for instances in which participants did not respond to any of the main trials during the verbal and spatial n-back training tasks. There were no instances of no responses during the WMT tasks. There were no instances of zero correct answers during the visual search and wordsearch active-control tasks. As the DSF/B started at a difficulty level that should be achievable by healthy participants who understood the task instructions and were engaged, we excluded participants who scored zero correct responses. Six participants were excluded from the DSF analysis as they scored zero at pre- (distributed-WMT: n=2, intensive-active-control: n=3) or post-training (distributed-active-control: n=1). No participant scored zero at both pre- and post-training. Four participants were excluded from the DSB analysis as they scored zero at pre- (distributed-WMT: n=1, distributed-active-control: n=1, intensive-active-control: n=1) or post-training (intensive-WMT: n=1). No participant scored zero at both pre- and post-training.

Comparison of simple linear regression and spline regression on smoothed data

A simple linear regression predicting mean n-back performance from performance at each time point was specified at group level. Additionally, at group level, learning rate was modelled using linear splines with one knot location at data point two (i.e., S2+S3). Linear splines were specified iteratively through to data point 14. The R-squared value of each model was compared and the model with the highest R-squared was selected as the spline regression model. The simple linear model and the spline model were then statistically compared using Akaike Information Criterion corrected (AICc) to determine whether the spline model was a significantly better fit than the simple linear model ($p=\exp((AIC_{spline} - AIC_{linear})/2)$. The spline regression model was a significantly better fit than the simple linear regression model for both WMT groups on the verbal and spatial n-back training tasks.

Table S1. Comparison of simple linear regression and spline regression.

	**Akaike Information Criterion corrected (AIC_c)**		***p***
	**Simple linear regression**	**Spline regression**	
Verbal n-back (DT: n=14; IT: n=18)			
Distributed-WMT	-24.47	-36.97	<.001
Intensive-WMT	-30.33	-53.25	<.001
Spatial n-back (DT: n=14; IT: n=16)			
Distributed-WMT	-18.91	-39.90	<.001
Intensive-WMT	-22.43	-43.99	<.001

Abbreviations: DT: distributed-WMT; IT: intensive-WMT.

Complementary analysis on smoothed data

Figure S5 displays WMT task performance at each smoothed time point as a function of training schedule (i.e., time point ‘1’ reflects the average of S1+S2 data, time point ‘2’ reflects the average of S2+S3 etc.)

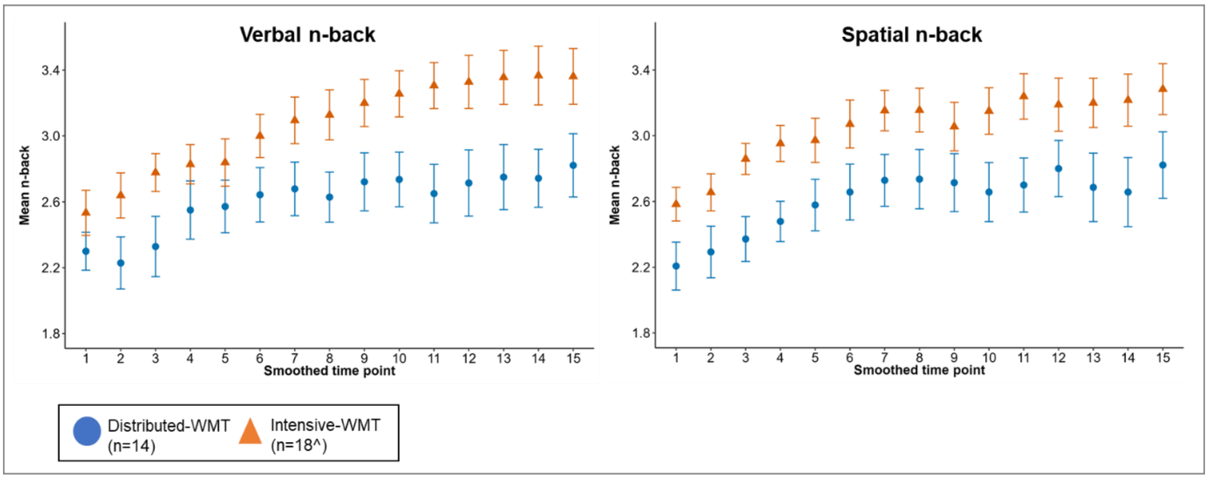

Figure S5. Smoothed verbal (left) and spatial (right) n-back training task performance as a function of training schedule (error bars represent standard error of the mean; ^ for the spatial n-back the 1vs3, 1vs4, 1vs6, and 1vs7 comparisons had n=17 in the intensive-WMT group due to excluded data for one participant at S4 and a different participant at S7).

We conducted a 2x2 ANOVA using mean n-back as the dependent variable, group (distributed-WMT/intensive-WMT) as between-subjects factors, and session (S1+S2/S15+S16) as a within-subjects factor **(Table S2).** Note: the active-control groups could not be included in this analysis as they only have data available for S1+S16. For completeness, the comparable statistical analyses on raw data (i.e., unsmoothed) are reported in **Table S2.**

Verbal n-back: There was a significant main effect of session (p<.001), indicating significantly better performance at the end of the intervention relative to the start of the intervention across groups. The main effect of group and group x session interaction were non-significant.

Spatial n-back: There was a significant main effect of group (p<.05), indicating significantly better performance in the intensive-WMT group relative to the distributed-WMT group across sessions. There was a significant main effect of session (p<.001), indicating significantly better performance at the end of the intervention relative to the start of the intervention across groups. The group x session interaction was non-significant.

Table S2. WMT task performance: inferential statistics.

	ANOVA			
	F	***p***	η_p^2	BF_{10}
**Smoothed data** (DT: n=14; IT: n=18)				
Verbal n-back				
Group (distributed-WMT/intensive-WMT)	3.79	.061	.11	1.74
Session (S1+S2/S15+S16)	42.84	<.001***	.59	297.89
Group x session	2.21	.148	.07	0.52
Spatial n-back				
Group (distributed-WMT/intensive-WMT)	4.62	.040*	.13	2.98
Session (S1+S2/S15+S16)	53.02	<.001***	.64	255.30
Group x session	0.23	.638	.01	0.31
**Raw data** (DT: n=14; IT: n=18)				
Verbal n-back				
Group (distributed-WMT/intensive-WMT)	3.09	.089	.09	0.91
Session (first/final)	31.20	<.001***	.51	1050.53
Group x session	1.06	.312	.03	0.48
Spatial n-back				
Group (distributed-WMT/intensive-WMT)	3.92	.057	.12	1.67
Session (first/final)	58.47	<.001***	.66	1972.84
Group x session	0.35	.561	.01	0.34

**p<.05, ***p<.001. Abbreviations: BF_{10} = Bayes Factor, BF>1 indicates evidence in favour of the alternative hypothesis; DT: distributed-WMT; IT: intensive-WMT.*

We conducted a 2x2 ANOVA, with mean n-back as the dependent variable, group (distributed-WMT/intensive-WMT) as a between-subjects factor, and session (S1+S2/SN*) as a within-subjects factor (SN refers to each subsequent session after S1+S2, i.e., 14 ANOVAs for each task; **Table S3**). *P*-values for group, session, and session x group effects were corrected for multiple comparisons using a 5% false discovery rate (FDR).

Verbal n-back: Performance at S1+S2 was significantly different from S4+S5 through to S15+16 across groups (i.e., a main effect of session). These *p*-values survived FDR correction. No group or group x session effects survived FDR correction.

Spatial n-back: Performance at S1+S2 was significantly different from S3+S4 through to S15+16 across groups (i.e., a main effect of session). These *p*-values survived FDR correction. Performance was significantly better across sessions in the intensive-WMT group relative to the distributed-WMT group for the S1+S2 vs. S5+S6 and S1+S2 vs. S15+S16 comparisons (i.e., a main effect of group). No group x session effects were significant.

Table S3. ANOVAs comparing WMT task performance at S1+S2 to each subsequent session.

	**Group (distributed-WMT / intensive-WMT)**				**Session (SA/SN)**				**Session x group**			
	F	***p***	η_p^2	**BF10**	***F***	***p***	η_p^2	**BF10**	***F***	***p***	η_p^2	**BF10**
Verbal												
1+2 vs. 2+3	2.89	.100	.09	2.45	0.09	.762	.00	0.26	2.52	.123	.08	0.30
1+2 vs. 3+4	3.57	.068	.11	3.12	3.08	.089	.09	0.41	1.93	.175	.06	0.35
1+2 vs. 4+5	1.94	.174	.06	1.02	15.28	<.001*	.34	1.30	0.10	.752	.00	0.27
1+2 vs. 5+6	1.86	.183	.06	0.90	12.65	<.001*	.30	1.30	0.04	.835	.00	0.27
1+2 vs. 6+7	2.71	.110	.08	1.38	24.75	<.001*	.45	8.32	0.58	.453	.02	0.32
1+2 vs. 7+8	3.14	.087	.10	1.66	32.16	<.001*	.52	21.55	1.21	.279	.04	0.38
1+2 vs. 8+9	4.13	.051	.12	2.61	24.79	<.001*	.45	17.15	2.06	.162	.06	0.47
1+2 vs. 9+10	3.84	.059	.11	1.96	31.77	<.001*	.51	65.85	1.61	.214	.05	0.45
1+2 vs. 10+11	4.47	.043	.13	2.50	38.15	<.001*	.56	154.65	2.34	.137	.07	0.53
1+2 vs. 11+12	5.89	.021	.16	5.35	37.33	<.001*	.55	79.97	5.28	.029	.15	0.81
1+2 vs. 12+13	4.38	.045	.13	2.78	41.76	<.001*	.58	82.45	4.13	.051	.12	0.64
1+2 vs. 13+14	4.48	.043	.13	2.55	38.99	<.001*	.57	144.08	3.34	.078	.10	0.63
1+2 vs. 14+15	4.83	.036	.14	2.78	34.68	<.001*	.54	145.43	3.25	.082	.10	0.67
1+2 vs. 15+16	3.79	.061	.11	1.74	42.84	<.001*	.59	297.89	2.21	.148	.07	0.52
Spatial												
1+2 vs. 2+3	4.70	.038	.14	15.39	1.71	.201	.05	0.33	0.01	.912	.00	0.26
1+2 vs. 3+4	7.82	.009	.21	40.02	11.28	.002*	.28	0.88	0.56	.459	.02	0.29
1+2 vs. 4+5	7.38	.011	.20	24.27	26.37	<.001*	.48	3.28	0.45	.508	.02	0.30
1+2 vs. 5+6	4.68	.039*	.14	5.46	32.31	<.001*	.52	5.39	0.02	.897	.00	0.27
1+2 vs. 6+7	4.96	.034	.15	3.90	41.89	<.001*	.59	15.37	0.00	.983	.00	0.28
1+2 vs. 7+8	5.97	.021	.17	4.79	60.28	<.001*	.68	115.34	0.00	.953	.00	0.29
1+2 vs. 8+9	5.03	.032	.14	3.93	40.41	<.001*	.57	80.36	0.06	.803	.00	0.29
1+2 vs. 9+10	3.91	.057	.12	2.41	29.26	<.001*	.49	21.56	0.04	.848	.00	0.26
1+2 vs. 10+11	5.99	.020	.17	6.47	29.16	<.001*	.49	30.84	0.38	.540	.01	0.32
1+2 vs. 11+12	6.75	.014	.18	8.94	47.98	<.001*	.62	134.47	0.96	.334	.03	0.37
1+2 vs. 12+13	4.18	.050	.12	2.46	43.96	<.001*	.59	125.71	0.01	.944	.00	0.28
1+2 vs. 13+14	5.44	.027	.15	4.93	30.51	<.001*	.50	34.68	0.49	.492	.02	0.33
1+2 vs. 14+15	5.70	.024	.16	5.97	29.89	<.001*	.50	26.24	0.86	.362	.03	0.36
1+2 vs. 15+16	4.62	.040*	.13	2.98	53.02	<.001*	.64	255.30	0.23	.638	.01	0.31

**Survived FDR correction. Abbreviations: BF_{10}=Bayes Factor, BF>1 evidence in favour of the alternative hypothesis; SA refers to the average of S1+S2, whilst SN refers to each subsequent session after SA.*

Table S4. Comparison of demographic and performance measures between groups at baseline.

	**Group**				**ANOVA or chi-square test**	
	**Distributed-WMT**	**Intensive-WMT**	**Distributed-active-control**	**Intensive-active-control**	***F* or χ2**	***p***
Age in years (M±SD (range))	65.93±4.97 (59-74)	65.00±5.35 (56-74)	68.06±8.26 (57-85)	65.56±6.87 (55-82)	0.72	.543
Gender (male/female) (count)	3/11	4/14	4/13	6/12	0.84	.839
Highest qualification achieved (count)					20.43	.309
- Degree: Master's or above	5	5	5	5		
- Degree: Undergraduate	2	9	4	7		
- A-level or equivalent	3	0	2	2		
- O-level or equivalent	2	2	2	1		
- No educational qualifications	0	0	0	2		
- Other	2	2	4	0		
- Prefer not to say	0	0	0	1		
Verbal n-back (mean n-back) (M±SD)	2.26±0.40 (1.60-3.00)	2.46±0.68 (1.20-3.60)	2.22±0.44 (1.40-2.80)	2.31±0.43 (1.40-3.40)	0.71	.553
Spatial n-back (mean n-back) (M±SD)	2.14±0.56 (1.40-3.20)	2.50±0.55 (1.60-3.40)	2.39±0.45 (1.60-3.00)	2.21±0.74 (1.00-3.80)	1.26	.295
DSF (span) (M±SD)	6.50±1.09 (5-9)	6.50±1.62 (3-9)	5.94±1.34 (3-8)	6.47±1.85 (3-9)	0.52	.672
DSB (span) (M±SD)	5.00±1.53 (3-8)	6.06±1.39 (4-8)	5.88±1.20 (4-8)	5.29±1.96 (2-8)	1.53	.217
Reasoning[1] (correct) (M±SD)	12.14±2.14 (8-15)	14.78±3.14 (10-20)	14.82±4.23 (7-24)	13.22±4.04 (5-24)	2.11	.108

Group differences reflect ANOVA values for age, digit-span forward, digit-span backward, relational reasoning, verbal n-back and spatial n-back, and chi-square values for gender and highest qualification achieved (the number of participants eligible for inclusion in each analysis differs slightly depending on the outcome measure). [1]*There was one extreme outlier in the intensive-active-control group. When this outlier was removed the p-value was significant (p=.037), but Tukey's honestly significant difference (HSD) post-hoc test showed no significant differences between groups. Abbreviations: M=mean; SD: standard deviation.*

Table S5. WMT and transfer task performance: descriptive statistics.

	Group			
	**Distributed-WMT (M±SD)**	**Intensive-WMT (M±SD)**	**Distributed-active-control (M±SD)**	**Intensive-active-control (M±SD)**
Verbal n-back				
First session	2.26±0.40	2.46±0.68	2.22±0.44	2.31±0.43
Final session	2.90±0.84	3.39±0.70	2.34±0.49	2.13±0.65
Difference (final-first)	+0.64	+0.93	+0.12	-0.18
Spatial n-back				
First session	2.14±0.56	2.50±0.55	2.39±0.45	2.21±0.74
Final session	2.91±0.81	3.40±0.73	2.38±0.55	2.51±0.70
Difference (final-first)	+0.77	+0.90	-0.01	+0.30
DSF				
Pre-training	6.50±1.09	6.50±1.62	5.94±1.34	6.47±1.85
Post-training	6.08±1.31	7.00±1.72	6.50±1.27	6.73±1.71
Difference (Post-Pre)	-0.42	+0.50	+0.56	+0.26
DSB				
Pre-training	5.00±1.53	6.06±1.39	5.88±1.20	5.29±1.96
Post-training	5.31±1.60	5.71±1.49	5.25±1.18	5.18±1.70
Difference (Post-Pre)	+0.31	-0.35	-0.63	-0.11
Relational reasoning				
Pre-training	12.14±2.14	14.78±3.14	14.82±4.23	13.22±4.04
Post-training	13.43±3.55	15.94±5.20	17.06±3.05	16.83±5.47
Difference (Post-Pre)	+1.29	+1.16	+2.24	+3.61

Table S6. ANOVAs comparing WMT task performance at S1 to each subsequent session.

	Group (distributed-WMT / intensive-WMT)				Session (S1/SN)				Session x group			
	F	*p*	η_p^2	BF_{10}	*F*	*p*	η_p^2	BF_{10}	*F*	*p*	η_p^2	BF_{10}
Verbal												
1 vs. 2	1.58	.218	.05	0.67	1.00	.324	.03	0.33	0.08	.774	.00	0.27
1 vs. 3	3.76	.062	.11	2.71	0.09	.762	.00	0.27	2.52	.123	.08	0.44
1 vs. 4	2.24	.145	.07	0.86	8.73	.006*	.23	2.65	0.37	.549	.01	0.32
1 vs. 5	1.04	.316	.03	0.52	11.37	.002*	.28	1.34	0.00	.952	.00	0.26
1 vs. 6	2.14	.154	.07	0.78	9.54	.004*	.24	3.92	0.25	.621	.01	0.31
1 vs. 7	2.43	.129	.08	1.01	33.72	<.001*	.53	54.41	1.06	.312	.03	0.39
1 vs. 8	2.82	.104	.09	1.07	17.46	<.001*	.37	20.19	0.96	.336	.03	0.41
1 vs. 9	3.97	.055	.12	1.95	20.50	<.001*	.41	24.38	2.40	.132	.07	0.59
1 vs. 10	2.40	.132	.07	0.78	29.53	<.001*	.50	295.63	0.65	.428	.02	0.39
1 vs. 11	5.02	.033	.14	3.09	33.22	<.001*	.53	104.50	4.59	.040	.13	0.86
1 vs. 12	4.71	.038	.14	2.47	30.83	<.001*	.51	121.81	4.09	.052	.12	0.85
1 vs. 13	2.99	.094	.09	1.25	38.33	<.001*	.56	129.10	2.64	.115	.08	0.55
1 vs. 14	5.33	.028	.15	1.91	21.92	<.001*	.42	258.56	2.26	.144	.07	0.77
1 vs. 15	3.69	.064	.11	1.47	28.32	<.001*	.49	144.97	2.34	.136	.07	0.62
1 vs. 16	3.09	.089	.09	0.91	31.20	<.001*	.51	1050.53	1.06	.312	.03	0.48
Spatial												
1 vs. 2	4.73	.038	.14	5.74	2.77	.106	.09	0.41	0.05	.831	.00	0.26
1 vs. 3	3.97	.055	.12	2.40	1.71	.201	.05	0.40	0.01	.912	.00	0.26
1 vs. 4	7.50	.010	.21	18.16	20.18	<.001*	.41	6.38	1.80	.190	.06	0.39
1 vs. 5	3.25	.081	.10	1.72	11.22	.002*	.27	2.46	0.04	.851	.00	0.26
1 vs. 6	3.95	.056	.12	2.13	18.74	<.001*	.39	13.44	0.21	.652	.01	0.31
1 vs. 7	3.39	.076	.11	1.40	38.98	<.001*	.57	23.26	0.37	.550	.01	0.26
1 vs. 8	5.20	.030	.15	2.82	38.30	<.001*	.56	473.77	0.38	.545	.01	0.35
1 vs. 9	3.13	.087	.09	1.58	34.80	<.001*	.54	33.72	0.01	.946	.00	0.27
1 vs. 10	3.12	.087	.09	1.28	22.32	<.001*	.43	32.29	0.01	.938	.00	0.27
1 vs. 11	7.37	.011	.20	8.90	27.57	<.001*	.48	62.09	1.68	.205	.05	0.48
1 vs. 12	4.06	.053	.12	1.96	50.00	<.001*	.63	567.97	0.13	.718	.00	0.31
1 vs. 13	2.91	.099	.09	1.09	23.19	<.001*	.44	50.41	0.00	.972	.00	0.27
1 vs. 14	7.01	.013	.19	6.17	19.08	<.001*	.39	33.59	1.37	.251	.04	0.48
1 vs. 15	3.50	.071	.10	1.61	25.53	<.001*	.46	41.21	0.11	.746	.00	0.30
1 vs. 16	3.92	.057	.12	1.67	58.47	<.001*	.66	1972.84	0.35	.561	.01	0.34

**Survived FDR correction. Abbreviations: BF_{10}=Bayes Factor, BF>1 evidence in favour of the alternative hypothesis; SN: refers to each subsequent session after S1.*

Table S7. Comparison of demographic information between participants who completed the study and participants who did not.

	**Completed the study (n=71)**	**Did not complete the study (n=8)**	***t*-test or chi-square test**	
			***t* or χ2**	***p***
Age[1] in years (M±SD (range))	66.20±6.68 (55-85)	63.13±7.45 (56-77)	1.22	.226
Gender (male/female) (count)	19/52	2/6	0.01	.915
Highest qualification achieved (count)			2.76	.838
- Degree: Master's degree or above	23	3		
- Degree: Undergraduate degree	22	4		
- A-level or equivalent	7	0		
- O-level or equivalent	7	0		
- No educational qualifications	2	0		
- Other	9	1		
- Prefer not to say	1	0		

[1] *Non-parametric Mann-Whitney U test on age also shows a non-significant difference in age between participants who completed the study and those who did not complete the study (p=.169).*

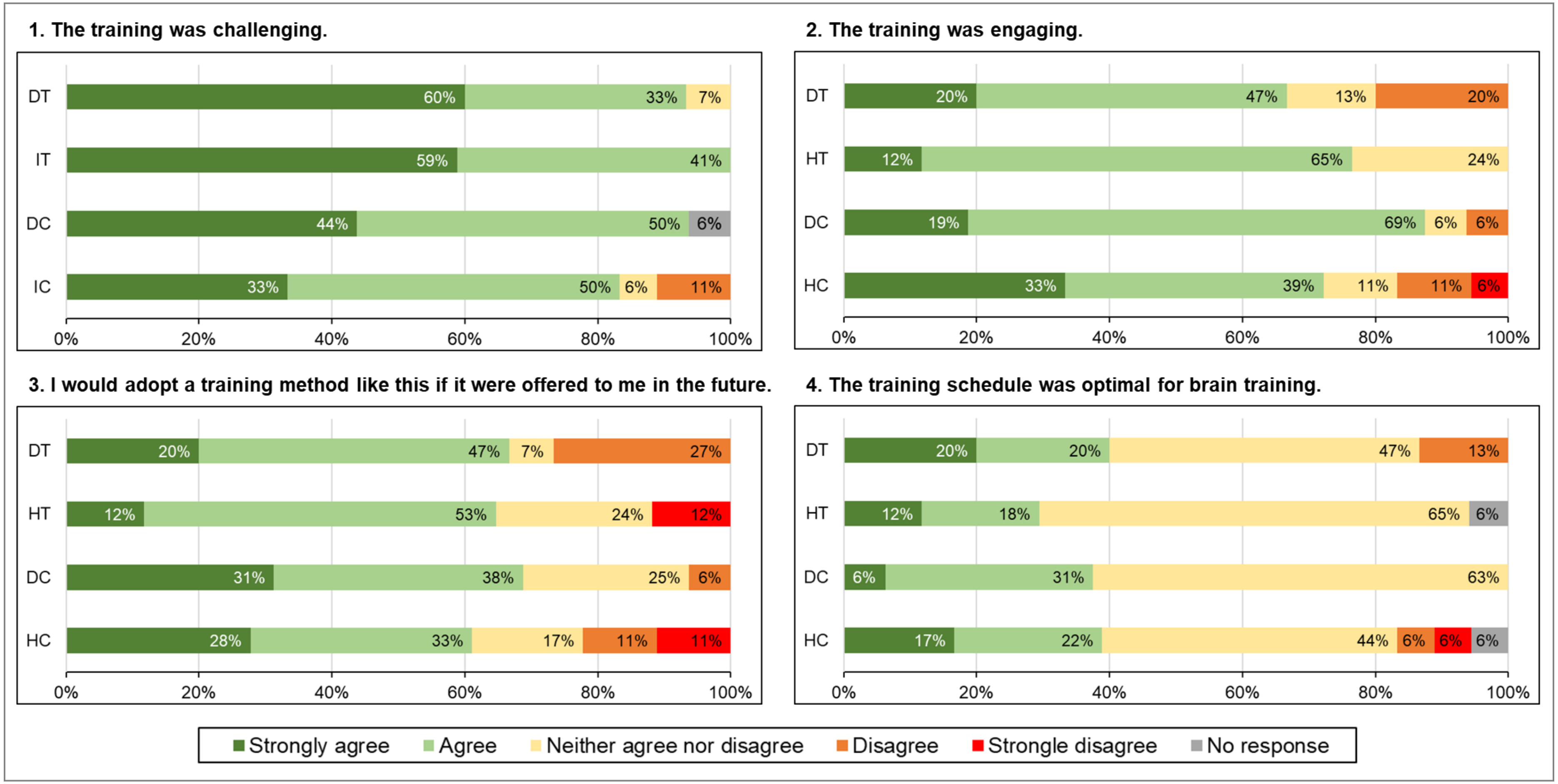

Figure S6. Questionnaire responses (abbreviations: DT: distributed-WMT; IT: intensive-WMT; DC: distributed-active-control; IC: intensive-active-control).

Table S8. Comparison of questionnaire responses.

	ANOVA		
	F	***p***	η_p^2
(1) The training was challenging			
(DT: n=15; IT: n=17; DC: n=15; IC: n=18)			
Treatment (WMT/active-control)	3.10	.083	.05
Schedule (distributed/intensive)	1.10	.299	.02
Treatment x schedule	1.88	.176	.03
(2) The training was engaging			
(DT: n=15; IT: n=17; DC: n=16; IC: n=18)			
Treatment (WMT/active-control)	0.38	.538	.01
Schedule (distributed/intensive)	0.01	.915	.00
Treatment x schedule	0.70	.408	.01
(3) I would adopt a training method like this if it were offered to me in the future			
(DT: n=15; IT: n=17; DC: n=16; IC: n=18)			
Treatment (WMT/active-control)	0.04	.843	.00
Schedule (distributed/intensive)	0.54	.465	.01
Treatment x schedule	0.62	.435	.01
(4) The training schedule was optimal for brain training			
(DT: n=15; IT: n=16; DC: n=16; IC: n=17)			
Treatment (WMT/active-control)	0.02	.900	.00
Schedule (distributed/intensive)	0.02	.900	.00
Treatment x schedule	0.00	.994	.00

Abbreviations: DT: distributed-WMT; IT: intensive-WMT.

References

Aichele, S., Cekic, S., Rabbitt, P., & Ghisletta, P. (2021). Cognition-mortality associations are more pronounced when estimated jointly in longitudinal and time-to-event models. *Frontiers in Psychology*, 3277. https://doi.org/10.3389/fpsyg.2021.708361

Aichele, S., Ghisletta, P., Corley, J., Pattie, A., Taylor, A. M., Starr, J. M., & Deary, I. J. (2018). Fluid intelligence predicts change in depressive symptoms in later life: The Lothian birth cohort 1936. *Psychological Science*, *29*(12), 1984–1995. https://doi.org/10.1177/0956797618804501

Bilker, W. B., Hansen, J. A., Brensinger, C. M., Richard, J., Gur, R. E., & Gur, R. C. (2012). Development of abbreviated nine-item forms of the Raven's standard progressive matrices test. *Assessment*, *19*(3), 354-369. https://doi.org/10.1177/1073191112446655

Borella, E., Carretti, B., Zanoni, G., Zavagnin, M., & De Beni, R. (2013). Working memory training in old age: an examination of transfer and maintenance effects. *Archives of Clinical Neuropsychology*, *28*(4), 331-347. https://doi.org/10.1093/arclin/act020

Chierchia, G., Fuhrmann, D., Knoll, L. J., Pi-Sunyer, B. P., Sakhardande, A. L., & Blakemore, S. J. (2019). The matrix reasoning item bank (MaRs-IB): novel, open-access abstract reasoning items for adolescents and adults. *Royal Society Open Science*, *6*(10), 190232. https://doi.org/10.1098/rsos.190232

Hebart, M, N., Dickter, A, H., Kidder, A., Kwok, W, Y., Corriveau, A., Van Wicklin, C., & Baker, C. I. (2019). THINGS: A database of 1,854 object concepts and more than 26,000 naturalistic object images. *PLOS ONE 14*(10), e0223792. https://doi.org/10.1371/journal.pone.0223792

Long, B., Yu, C. P., & Konkle, T. (2018). Mid-level visual features underlie the high-level categorical organization of the ventral stream. *Proceedings of the National Academy of Sciences*, *115*(38), E9015-E9024. https://doi.org/10.1073/pnas.1719616115

Raven, J., & Raven, J. (2003). Raven Progressive Matrices. In R. S. McCallum (Ed), *Handbook of Nonverbal Assessment* (pp. 223–237). Kluwer Academic/Plenum Publishers. https://doi.org/10.1007/978-1-4615-0153-4_11
